# Disproportionate Contribution of Riparian Inputs to Organic Carbon Pools in Freshwater Systems

**DOI:** 10.1007/s10021-014-9772-6

**Published:** 2014-04-29

**Authors:** Trent R. Marwick, Alberto Vieira Borges, Kristof Van Acker, François Darchambeau, Steven Bouillon

**Affiliations:** 1 Department of Earth and Environmental Sciences, Katholieke Universiteit Leuven (KU Leuven), Celestijnenlaan 200E, 3001 Leuven, Belgium; 2 Chemical Oceanography Unit, University of Liège (ULg), Institut de Physique (B5), 4000 Liège, Belgium

**Keywords:** Riverine organic carbon, Carbon stable isotopes (δ^13^C), Tropical rivers, C_4_, C_3_, Riparian vegetation, Madagascar

## Abstract

A lack of appropriate proxies has traditionally hampered our ability to distinguish riverine organic carbon (OC) sources at the landscape scale. However, the dissection of C_4_ grasslands by C_3_-enriched riparian vegetation, and the distinct carbon stable isotope signature (δ^13^C) of these two photosynthetic pathways, provides a unique setting to assess the relative contribution of riparian and more distant sources to riverine C pools. Here, we compared δ^13^C signatures of bulk sub-basin vegetation (δ^13^C_VEG_) with those of riverine OC pools for a wide range of sites within two contrasting river basins in Madagascar. Although C_3_-derived carbon dominated in the eastern Rianala catchment, consistent with the dominant vegetation, we found that in the C_4_-dominated Betsiboka basin, riverine OC is disproportionately sourced from the C_3_-enriched riparian fringe, irrespective of climatic season, even though δ^13^C_VEG_ estimates suggest as much as 96% of vegetation cover in some Betsiboka sub-basins may be accounted for by C_4_ biomass. For example, δ^13^C values for river bed OC were on average 6.9 ± 2.7‰ depleted in ^13^C compared to paired estimates of δ^13^C_VEG_. The disconnection of the wider C_4_-dominated basin is considered the primary driver of the under-representation of C_4_-derived C within riverine OC pools in the Betsiboka basin, although combustion of grassland biomass by fire is likely a subsidiary constraint on the quantity of terrestrial organic matter available for export to these streams and rivers. Our findings carry implications for the use of sedimentary δ^13^C signatures as proxies for past forest-grassland distribution and climate, as the C_4_ component may be considerably underestimated due to its disconnection from riverine OC pools.

**Electronic supplementary material:**

The online version of this article (doi:10.1007/s10021-014-9772-6) contains supplementary material, which is available to authorized users.

## Introduction

The riparian zone plays a key role in regulating the delivery of solutes to the adjoining inland surface waters (Remington and others 2007; Ranalli and Macalady 2010; Bouwman and others 2013). However, determining the origin of riverine carbon (C) at the landscape scale is often hampered by a lack of appropriate proxies distinguishing various landscape units and their constituent organic carbon (OC) pools. Riverine OC pools constitute an amalgam of allochthonous (for example, vegetation and soils) and autochthonous (for example, phytoplankton, benthic algae, aquatic macrophytes) C sources, with the relative contributions of each to riverine OC pools increasingly disentangled through multi-proxy approaches, such as the use of C stable isotopes (δ^13^C) in combination with elemental (for example, C:N) and particulate OC (POC):Chlorophyll *a* (Chl*a*) ratios (Hamilton and Lewis 1992; Kendall and others 2001; Bernardes and others 2004; Finlay and Kendall 2007; Tamooh and others 2012), and more recently the incorporation of GIS-based toolsets (Ballester and others 2013).

In freshwaters with negligible algal production, the predominant driver of riverine OC δ^13^C signatures is the proportion of organic matter (OM) derived from terrestrial vegetation following the C_3_ photosynthetic pathway (woody plants and trees, temperate grasses; δ^13^C ~ −27‰) compared to the less fractionating C_4_ photosynthetic pathway (largely tropical and subtropical grasses; δ^13^C ~ −13‰) (Hedges and others 1986; Bird and others 1994).

Following from these observations, it has been suggested that marine sedimentary sequences adjacent to major river mouths in the tropics may store extensive records of basin vegetation evolution in the form of terrestrial bulk OC δ^13^C signatures (Mariotti and others 1991). Indeed, others have employed stable isotope techniques to reconstruct paleo-vegetation distribution and paleo-climate regime from OM buried within various environments, including lacustrine and fluvial deposits (Cerling and others 1988; Mora and others 2002) as well as nearshore (Santschi and others 2007) and offshore (dos Santos and others 2013) marine deposits. Yet, such studies often assume no inherent differences in sediment entrainment or transport between C_3_- and C_4_-derived carbon from source to sink (Wynn and Bird 2007). In fact, notwithstanding the considerable contribution of C_4_ biomass to sub-Saharan vegetation cover (~31%; Still and Powell 2010), few studies have identified substantial contribution from C_4_-derived C in riverine POC or dissolved OC (DOC) pools of grasslands or savannah grasslands (Mariotti and others 1991; Bird and others 1992; Bird and Pousai 1997; Wynn and Bird 2007; Tamooh and others 2012). The predominantly perennial connection of the C_3_-enriched riparian zone to active (surface and ground-) water channels, relative to the seasonally parched and disconnected C_4_-dominated grasslands, has been identified as a key driver of riverine organic C pools within subtropical and tropical savannah/grassland ecosystems globally (for example, Congo [Mariotti and others 1991]; Amazon [Bird and others 1992]; Australian rivers [Bird and Pousai 1997]; Cameroonian rivers [Bird and others 1994, 1998]).

Despite these findings, little systematic analysis of the proportional contribution of these landscape units to riverine organic C pools has been conducted (for example, Ballester and others 2013). Biomes with distinct spatial segregation of the C_3_ and C_4_ photosynthetic pathways should provide a unique scenario with which to assess the proportional contribution of different landscape units to riverine C pools with the use of C stable isotopes. Such settings, for example, include tropical and subtropical C_4_-dominant *grasslands* (*grasslands* as defined by Torello-Raventos and others (2013)) partially disconnected from adjacent streams and rivers by a comparatively C_3_-enriched riparian fringe (for example, see Mariotti and others 1991; Bird and others 1994).

A region where the spatial partitioning of C_3_ and C_4_ biomass is astoundingly apparent is within the Hauts-Plateaux grasslands and savannahs of central Madagascar, with estimates that greater than 75% of the island’s surface area is covered by these vegetation biomes (Bond and others 2008). Previous research (Ralison and others 2008) conducted in the Betsiboka basin, which drains much of the Hauts-Plateaux region, reports that C of C_4_ origin accounts for 30% of the riverine DOC and POC pools in the upper part of the estuary, which was considerably lower than expected given estimates that C_4_ vegetation comprised more than 80% of basin vegetation land cover. Here, we build on their observations by exploring δ^13^C of riverine POC (δ^13^C_POC_) and DOC (δ^13^C_DOC_) as a function of bulk sub-basin vegetation δ^13^C (δ^13^C_VEG_) and soil δ^13^C signatures in the Betsiboka basin. For comparison, we also report results for an eastern Malagasy drainage basin, the Rianila, characterised by C_3_ rainforest.

## Materials and Methods

### Basin Characteristics

#### The Betsiboka Basin

**Figure 1:**
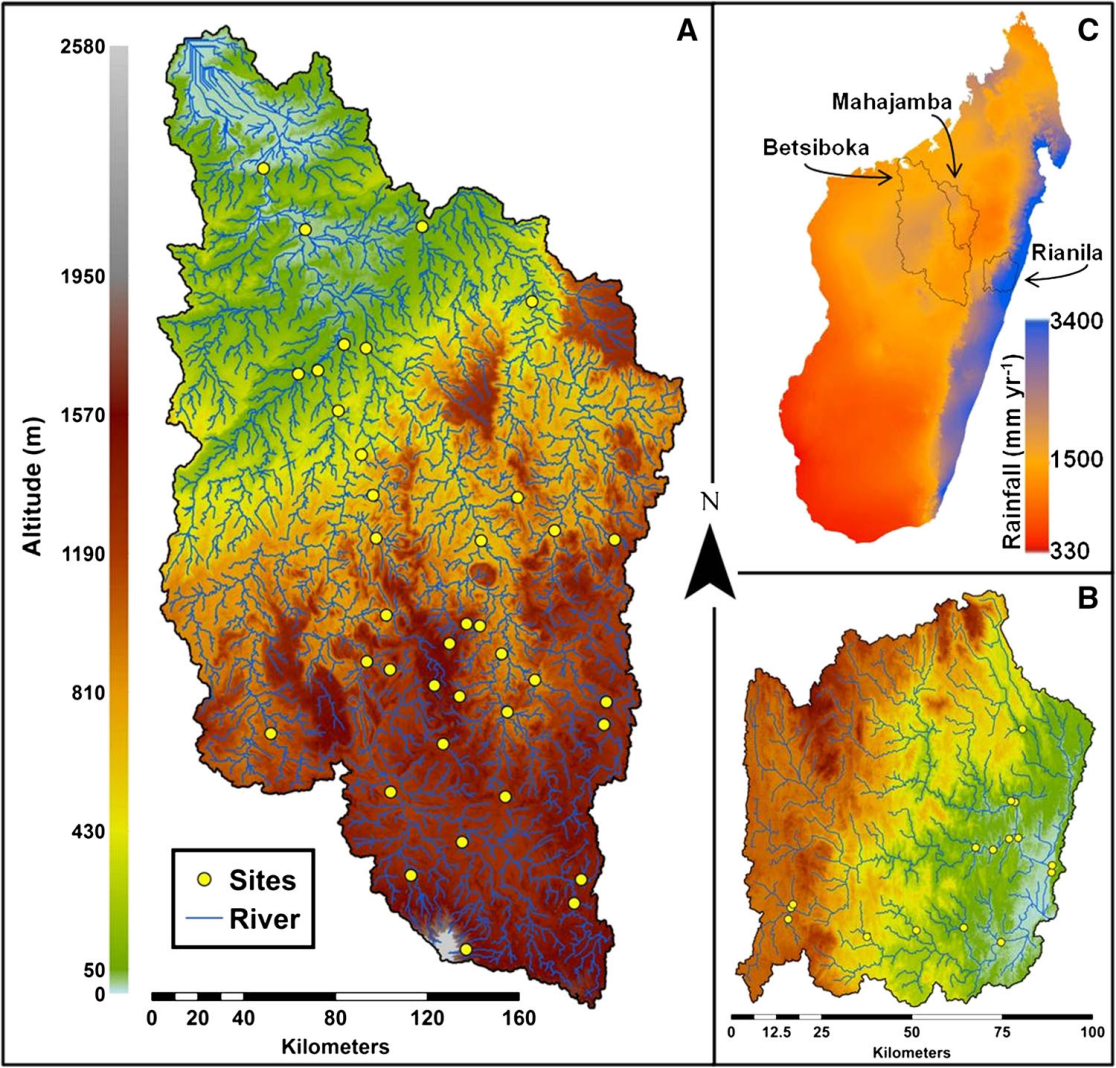
Digital elevation models (DEM) of the two Madagascan study basins: **A** Betsiboka, and **B** Rianila. **C** Shows the strong disparity of annual precipitation between the eastern and western regions of the island. The Mahajamba basin (**C**) experiences a sporadic connection with the Kamoro River (Betsiboka basin) during elevated discharge periods.

**Figure 2:**
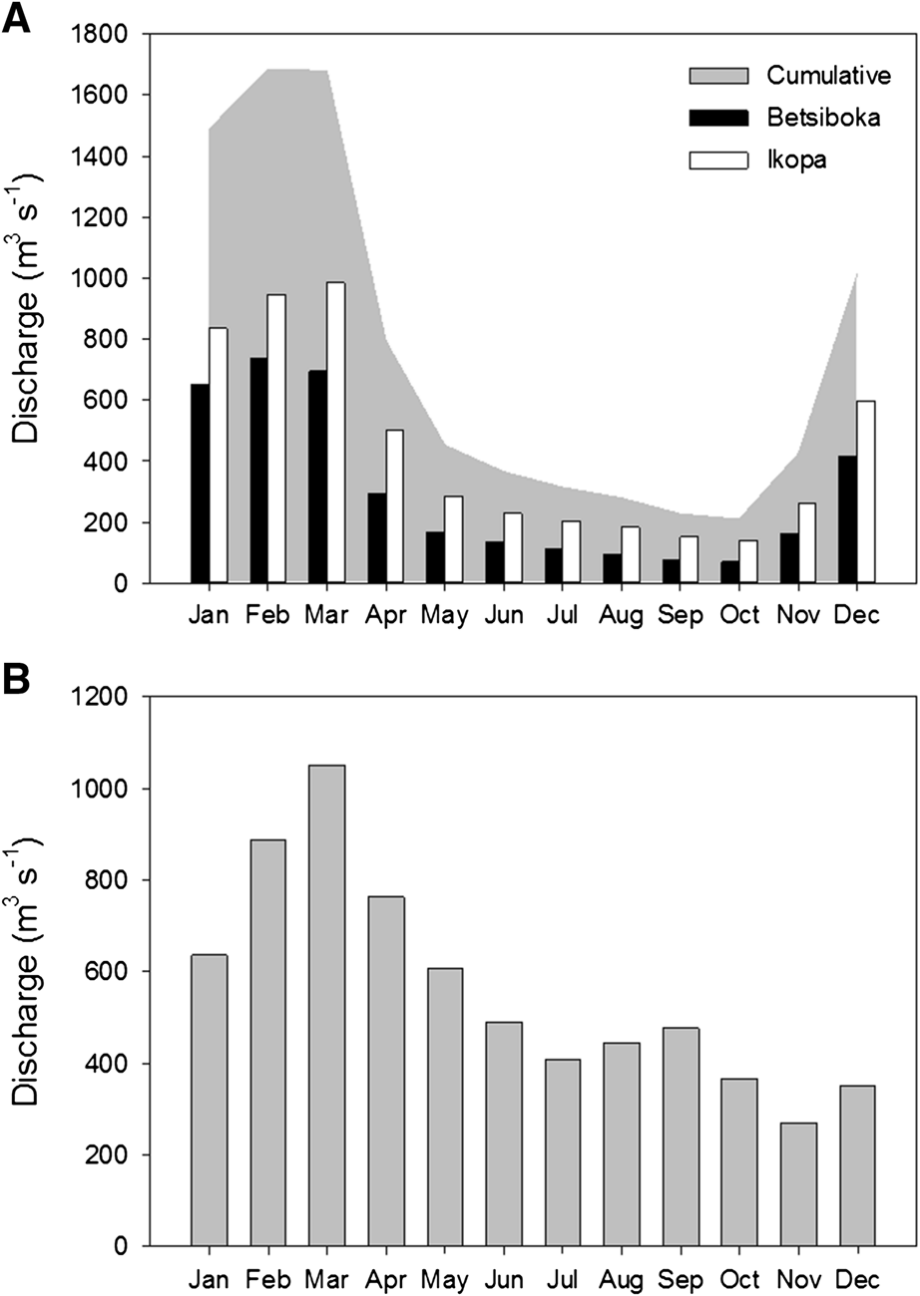
Overview of available historical mean monthly discharge data for **A** the Betsiboka (at Ambodiroka) and Ikopa (at Bevomanga) rivers, and **B** the Rianila at Brickaville. No long-term gauging stations have been established below the junction of the Ikopa with the Betsiboka (~180 km upstream of the outlet) due to the mobile nature of the main channel through the poorly consolidated floodplain sediments, and the wide dispersal of floodwaters over the low lying floodplains following bankfull discharge. Cumulative discharge in **A** only represents the sum of Betsiboka and Ikopa discharge upstream of their confluence, and excludes any contribution from the perennial Kamoro River, or from the Mahajamba basin when it is intermittently connected during elevated discharge conditions. Data for the Rianila do not include contributions from the Iaroka River, which joins downstream of Brickaville and drains approximately 1,300 km^2^ of the Rianila basin. Data from Chaperon and others (1993).

The Betsiboka basin drains between 49,000 to 63,000 km^2^ of central and western Madagascar (Figure 1A), forming north of the Malagasy capital, Antananarivo, at the confluence of the Jabo and Amparihibe rivers (at 938 metres above sea level [m.a.s.l.]) in the Hauts-Plateaux. This region is often cited as one of the most erosive landscapes identified globally, with annual yields of 20,000 to 40,000 t km^−2^ (World Bank 1998; USAID 1998), although Cox and others (2009) suggest more modest yields of 32 t km^−2^ y^−1^ estimated from cosmogenic ^10^Be data of river sediments. *Lavakas* (meaning ‘holes’ in Malagasy) are mass failure features common to the Hauts-Plateaux which lead to considerable loss of arable land, elevated suspended and river bed sediment loads (Cox and others 2003, 2004, 2009), and subsequent alluviation of streams, rivers and estuaries (Zavada and others 2009). Relative to their limited spatial extent, *lavakas* contribute up to 84% of terrestrial sediment input to Malagasy rivers (Cox and others 2009). At 180 km downstream of the confluence with the Ikopa River, the principle tributary of the Betsiboka basin, the Betsiboka River flows into the Bombetoka estuary. Average discharge for the Betsiboka and Ikopa rivers, upstream of their confluence, is about 301 and about 77 m^3^ s^−1^, respectively (Figure 2A).

**Figure 3:**
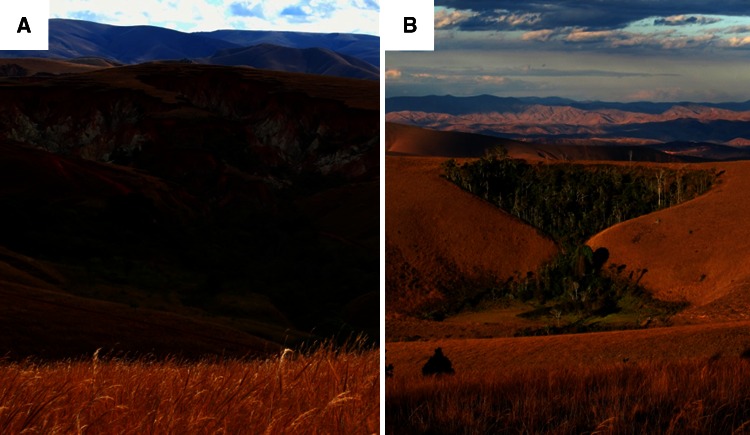
Examples of *lavakas* in the Hauts-Plateaux of central Madagascar. **A** With the collapse of the overlying lateritic soil layer, the more nutrient enriched saprolite layer becomes exposed. As *lavakas* evolve (**B**), often a C_3_ dominant vegetation complex develops in response to increased nutrient and water availability (photos T.R.M.).

Following the recent classification system proposed by Torello-Raventos and others (2013), the terms grasslands and savannah grasslands, with dominance of C_4_ grass species, would be appropriate vegetation classifiers for the Hauts-Plateaux region of the Betsiboka basin. Charcoal in regional stratigraphic records suggests natural development and maintenance of the Malagasy grasslands by fire (Burney 1987a, b, c), though the present fire regime is heavily influenced by anthropogenic practices such as pasture management for cattle grazing, with estimates of a quarter to a half of Malagasy grasslands (by land area) are burned annually (Kull 2012). Primary C_3_ vegetation is sparse throughout (approximately 4% of basin area; see Du Puy and Moat 1996, 1999), with C_3_-enriched riparian fringes scattered throughout the basin. During the mid-phase of *lavaka* evolution (Wells and others 1991; Wells and Andriamihaja 1993, 1997) a C_3_-enriched forest complex may establish within the *lavaka* in response to the localised micro-climate (Figure 3).

#### The Rianila Basin

The Rianila basin drains approximately 7,800 km^2^ and is an archetypal basin of the eastern slopes of Madagascar (Figure 1B), rising in the Betsimisaraka Heights at 1510 m.a.s.l. Du Puy and Moat (1996, 1999) approximate 61% of the Rianila basin is vegetated by low- or mid-latitude humid evergreen primary forest. Due to the north–south orientated ridge segregating eastern from central and western Madagascar, a rain-shadow effect results in considerably elevated annual rainfall in eastern regions relative to the rest of the island (Figure 1C). August to October tend to be the driest months, yet trade-winds from the southeast can provide consistent rainfall throughout these months. Annual precipitation ranges between 2,500 and 3,000 mm, resulting in an average discharge of 408 m^3^ s^−1^ at Brickaville (nt. excludes downstream contribution from the Iaroka basin) (Aldegheri 1972) (Figure 2B).

#### Sampling and Analytical Techniques

Sampling was conducted in the Betsiboka and Rianila basins between July and September 2010 (dry season) and January to February 2012 (wet season). Within the Betsiboka, 43 sites were sampled during the dry season with repeated samples at 33 sites in the wet season, whereas 16 sites were sampled in the Rianila basin during both seasons. Riverine physico-chemical parameters (pH, dissolved oxygen, conductivity) were measured using a combination of a YSI Professional Plus (Pro Plus, Quattro cable bulkhead) and YSI Professional Optical Dissolved Oxygen (Pro ODO) instruments. Calibrations for pH were performed daily using National Bureau of Standards (NBS) buffers of pH 4 and 7.

A Niskin bottle was used to gather riverine solute samples 0.5 m below the surface, or using a rope and bucket when sampling from bridges. For bulk POC concentrations and C stable isotope composition (δ^13^C_POC_), a known volume of surface water was filtered on 25 mm GF/F filters (pre-combusted overnight at 450°C; nominal porosity = 0.7 µm) and subsequently air dried. Later, filters were exposed to HCl fumes for at least 4 h to remove inorganic C (IC), subsequently re-dried and packed in Ag cups. Soil OC samples (0–5 cm layer) were collected within 50 m of the river, whereas river sediment OC samples were taken with a Van Veen grab. Soil and sediment samples were only gathered during the dry season. These samples were stored immediately in liquid N_2_ until return to the laboratory, where they were stored at −20°C. Samples were later dried and homogenised using a mortar and pestle. A weighed subsample was transferred into a Ag cup to which a 10% HCl solution was added to remove carbonates. Samples were then dried at 60°C for 24 h and, if necessary, the HCl procedure was repeated. Leaf litter samples were collected from select sites in each basin below vegetation visually representative of the area. At some sites in the Betsiboka, separate samples were collected from the riparian fringe and the surrounding C_4_ grasslands. Litter was air dried in the field and stored in air-tight zip-lock bags, and dried at 50°C for 24 h in the laboratory oven. Subsamples were ground and homogenised with a portion transferred to Ag cups ready for analysis. Bulk POC and δ^13^C_POC_, δ^13^C of soil (δ^13^C _SOIL_) and river bed sediments (δ^13^C_SED_), as well as leaf litter δ^13^C (δ^13^C_LITTER_) were determined on a Thermo elemental analyzer–isotope ratio mass spectrometer (EA-IRMS) system (FlashHT with DeltaV Advantage), and by monitoring the 44, 45 and 46 *m*/*z* signal on the IRMS. Quantification and calibration of δ^13^C data were performed with IAEA-C6 and acetanilide which are internally calibrated against international standards. Reproducibility of δ^13^C_POC_ measurements was typically better than ±0.2‰ whereas relative standard deviations for calibration standards for POC measurements are typically less than 2% and always less than 5%.

DOC samples were obtained by pre-filtering surface water through pre-combusted GF/F filters (0.7 µm), with further filtration through 0.2-µm syringe filters, and preserved with H_3_PO_4_ in glass vials with Teflon-coated screw caps. Bulk DOC and δ^13^C (δ^13^C_DOC_) were measured with either a customised Thermo HiperTOC coupled to a Delta + XL IRMS (Bouillon and others 2006), or by manual injection in a Thermo IsoLink HPLC-IRMS (similar to the method described in Albéric 2011).

Chl*a* was collected in the field following the procedure of POC above, and used as an index of phytoplankton biomass (Reynolds 2006). Total Chl*a* measurement was achieved by high performance liquid chromatography (HPLC) for dry season samples. The reproducibility of Chl*a* by HPLC has been demonstrated to be within 10% (Claustre and others 2004). Samples for HPLC analysis were obtained from filtration of 200–1,000 ml on Whatman GF/F filters, of 0.7 µm nominal pore size. Filters were immediately stored in liquid N_2_, and upon return to the lab were kept frozen (at −25°C) until further processing. Pigment extraction was carried out in 3 ml of 90% HPLC grade acetone. After two 15-min sonications separated by an overnight period at 4°C in the dark, pigment extracts were processed through a Waters HPLC system comprising a Waters 470 fluorescence detector setup for optimal detection of chlorophyll. The separation was achieved using a 25 cm Waters Nova-Pak C18 column and the ternary gradient of Wright (1991). Calibration was achieved using four-point calibration curves established with a Chl*a* standard purchased from DHI, Denmark. Wet season Chl*a* filters, from the Betsiboka basin only, were extracted with 1 ml of 90% acetone buffered with saturated MgCO_3_, after which they were incubated overnight at 4°C. Extracts were centrifuged at 10,000 rpm for 10 min, followed by measurement on a Perkin Elmer Spectrophotometer Lambda20 at 664 and 750 nm pre- and post-acidification with 750 µl of 0.1 M HCl. Wet season Chl*a* concentrations were calculated according to Lorenzen (1967). The majority of sites, however, were below the quantification limit of our methodology, hence we used the quantification limit to provide minimum estimates for POC:Chl*a* ratios (both expressed in mg C) for the wet season dataset. Chl*a* samples were not collected in the Rianila basin during the wet season due to the unavailability of liquid N_2_ for sample preservation.

In-situ pelagic primary production (PPP) was determined by filling two 500-ml polycarbonate bottles with surface water and adding 500 µl of a ^13^C-enriched bicarbonate solution (>99.8% ^13^C NaH^13^CO_3_, ~40 mg dissolved in 12 ml of surface water pre-filtered at 0.2 µm). Bottles were incubated instream under ambient light and temperature conditions for approximately 2 h, after which a subsample from each bottle was filtered as for POC above. Filters were later exposed to HCl fumes for 4 h to remove IC, re-dried and packed in Ag cups. A separate 12-ml glass headspace vial was filled for measuring dissolved IC δ ^13^C following enrichment (δ^13^C_DIC-PPP_) from each incubation bottle with the addition of 10 μl HgCl_2_ to inhibit further biological activity.

For the analysis of δ^13^C_DIC-PPP_, a 2 ml helium (He) headspace was created, and H_3_PO_4_ was added to convert all DIC species to CO_2_. After overnight equilibration, part of the headspace was injected in the stream of an elemental analyser–isotope ratio mass spectrometer (EA-IRMS; ThermoFinnigan FlashHT and ThermoFinnigan DeltaV Advantage). The output δ^13^C was corrected for isotopic equilibration between gaseous and dissolved CO_2_ as described in Gillikin and Bouillon (2007). Analysis of PPP filters followed the procedures outlined above for POC analysis. The PPP rates were calculated based on Hama and others (1983):

1 $$ {\text{PPP}} = \frac{{{\text{POC}}_{\text{f}} (\%^{13} {\text{POC}}_{\text{f}} - \%^{13} {\text{POC}}_{\text{i}} )}}{{t(\%^{13} {\text{DIC}} - \%^{13} {\text{POC}}_{\text{i}} )}}, $$

where POC_f_ is the particulate OC after incubation, %^13^POC_i_ and %^13^POC_f_ the initial and final (that is, after incubation) percentage ^13^C of the POC, *t* is the incubation time and %^13^DIC the percentage ^13^C of the DIC after the bottles had been spiked. It must be stressed that our PPP data are not depth integrated and are only valid for the upper surface waters, and should be considered to represent the maximum volumetric rates. They are mainly used here, in conjunction with Chl*a* measurements, to constrain an upper limit of autochthonous contribution to riverine OC pools.

All statistical analyses were performed in SigmaPlot v12. Where sites were measured in both dry and wet seasons, paired *t* tests (hereafter Pt–T) were used to assess significant seasonal changes in the concentrations and δ^13^C of the OC pools, as well as other biogeochemical parameters. Where the seasonal observations were not normally distributed, Pt–T were replaced by Wilcoxon signed rank tests (hereafter SRT).

## Results

### Carbon Stable Isotope Composition of Leaf Litter and Soils

The average δ^13^C of leaf litter OC (δ^13^C_LITTER_) was −30.2 ± 0.7‰ (*n* = 4) and −29.5 ± 1.1‰ (*n* = 6) in the Rianila basin and the riparian zone of the Betsiboka basin, respectively, agreeing with modelled δ^13^C of C_3_ vegetation for these regions based on Kohn (2010; −30.3 ± 0.3 and −29.3 ± 0.0‰, respectively). Likewise, our data are comparable with those of Crowley and others (2011), who report an average C_3_ δ^13^C_LITTER_ of −31.6 ± 1.1‰ in the eastern Malagasy rainforests and −28.2 ± 1.3‰ for C_3_ vegetation in the lower Betsiboka basin. The grasslands of the Betsiboka basin were clearly dominated by C_4_ species (average δ^13^C_LITTER_ −12.2 ± 0.7‰; *n* = 9).

These C_3_ and C_4_ end-members encapsulate the gradient observed in the δ^13^C of the soil OC (δ^13^C_SOC_) pool in the two basins. In the Betsiboka basin, the δ^13^C_SOC_ ranged between −25.8‰ (reflecting a predominantly C_3_ origin) and −12.0‰ (pure C_4_ origin), whereas SOC ranged from a pure C_3_ (−28.2‰) to mixed C_3_:C_4_ (−16.9‰) origin in the Rianila basin.

### Estimation of Bulk Sub-basin Vegetation δ^13^C (δ^13^C_VEG_) Signatures

Still and Powell (2010) provide a modelled C_4_ vegetation distribution map for the African continent and the island of Madagascar based on climate datasets, the MODIS Vegetation Continuous Fields (VCF) product (estimate of annual percent cover of herbaceous, tree and bare soil), and the Global Land Cover Map 2000, and converted this to an *isoscape* using vegetation δ^13^C end-members of −27‰ and −12% for pure C_3_ coverage and pure C_4_ vegetation, respectively.

With the use of the hydrology tool set of ArcMap 10 and SRTM Void Filled digital elevation data (3 arc-seconds; Earth Explorer, http://earthexplorer.usgs.gov/), we extracted the upstream contributing sub-basin at each sampling location from the Still and Powell (2010) crop-corrected output. Resultant bulk vegetation δ^13^C (δ^13^C_SP_) values were calculated as the average pixel value within the extracted sub-basin (see Supplementary Material). To permit comparison between the δ^13^C_SP_ estimates and riverine δ^13^C_OC_ pools, the δ^13^C_SP_ values were normalised to our δ^13^C_LITTER_ end-members (that is, Rianila: 100% C_3_ = −30.2‰, 100% C_4_ = −12.2‰ [C_4_ grasses from Betsiboka]; Betsiboka: 100% C_3_ = −29.5‰, 100% C_4_ = −12.2‰).

The δ^13^C_SP_ values were calculated from 28 sub-basins, with basin area ranging from 17 to 54,019 km^2^ and covering a δ^13^C spectrum of −30.0 to −17.6‰. As expected, the Rianila sub-basins were dominated by C_3_ vegetation, with an average bulk δ^13^C_SP_ value of −27.6 ± 1.7‰ (range −22.5 to −30.0‰, or 57–99% C_3_ cover, *n* = 16). The Betsiboka sub-basin average bulk δ^13^C_SP_ value (−18.7 ± 0.6‰, range −17.6 to −19.6‰, or 57 and 69% C_4_ vegetation cover, *n* = 12) was more mixed than those of the Rianila, though with less C_4_ influence than was expected from onsite observation.

As originally stipulated by Still and Powell (2010), their outputs were intended for continental analysis of C_4_ vegetation distribution. Our attempt here to apply them on a regional to local scale highlights one issue with the climatic constraint used to identify areas of potential C_4_ land cover. Model output is limited in the upper- to mid-basin of the Betsiboka, where a broad region identified in the field, and confirmed through satellite imagery, to be dominated by C_4_ grasslands has been classified as pure C_3_ by Still and Powell (2010). We believe this to be a factor of the perennially cool climate of the central highlands not meeting the C_4_ bioclimatic conditions constraining the model output. Differences in C_3_ and C_4_ quantum yield, the ratio of moles of CO_2_ assimilated relative to moles of photosynthetic active radiation absorbed by a leaf when photosynthesis is limited by low light conditions (Ehleringer and Björkman 1977; Collatz and others 1998), is a key control on the distribution and ecological sorting of the two photosynthetic pathways (Ehleringer 1978). Measured temperatures where the quantum yield of C_3_ and C_4_ photosynthesis are equivalent (that is, the crossover temperature), vary from of 16 to 24°C (Ehleringer and others 1997), with Still and Powell (2010) using a crossover temperature of 21°C. Yet Antananarivo, situated in the upper Betsiboka-Ikopa basin, experiences average annual monthly temperatures of no higher than 21°C, placing this region outside of the climatic constraints identifying areas of potential C_4_ land cover. We suggest that a lower crossover temperature may be required to adequately model regional C_4_ distribution in the Malagasy Hauts-Plateaux, where average monthly temperatures remain below 21°C year-round yet C_4_ grasslands spatially comprise a significant portion of basin vegetation cover.

**Figure 4:**
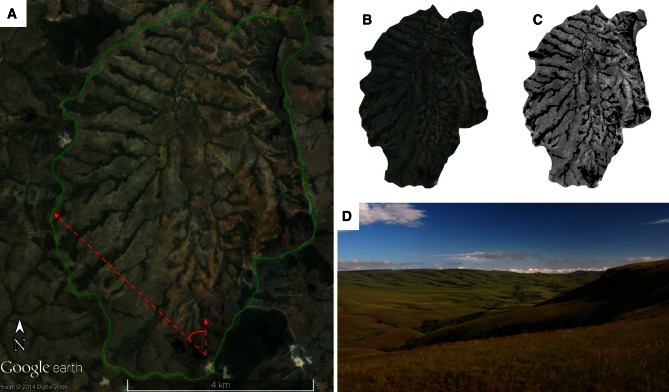
Illustration of the approach used to calculate δ^13^C_SBE_ values for select sub-basins of the Betsiboka basin. **A** Overview of the sub-basin of site B38 (36 km^2^), showing the sub-basin area (*green polygon*). **B** Site B38 sub-basin extracted and **C** converted to greyscale. C_3_ vegetation (δ^13^C_LITTER_ = −29.5‰) is classified by *black pixels*, with all other pixels classified as C_4_ vegetation (δ^13^C_LITTER_ = −12.2‰). **D** C_4_ grasses typically dominate the undulating valleys of the mid-basin Betsiboka, with C_3_ vegetation confined to the perennially moist soils of the riparian zone and old *lavaka* depressions (field-of-view is indicated by the *red arrows* in **A**). See Supplementary Materials for detailed techniques and similar examples for other sub-basins (Data sourced from Google Earth with data provided by DigitalGlobe).

For these reasons, we developed our own satellite image-based estimate of C_3_ and C_4_ distribution (δ^13^C_SBE_) for a selection of the Betsiboka sub-basins. Satellite imagery of 21 sub-basins was extracted from Google Earth Pro (see Supplementary Material). Full sub-basin area was extracted for eight sub-basins (for example, Figure 4), whereas variation in image quality and decreased image resolution at larger scales necessitated the need to extract singular or multiple quadrants representative of total vegetation for the remaining sub-basins (see Supplementary Material). Satellite imagery was subsequently converted to greyscale, followed by increases in gamma correction and contrast, with resultant black pixels classified as C_3_ cover (δ^13^C_LITTER_ = −29.5‰) and all remaining pixels classified as C_4_ cover (δ^13^C_LITTER_ = −12.2‰), with the sum average pixel value used to estimate the sub-basin δ^13^C_SBE_ signature.

Bulk δ^13^C_SBE_ estimates ranged between −22.8 and −12.9‰ (average δ^13^C_SBE_ 14.7 ± 2.2‰, *n* = 21). Where paired δ^13^C_SP_ and δ^13^C_SBE_ values were estimated, δ^13^C_SBE_ values were significantly (Pt–T: *P* < 0.001) enriched in ^13^C (+4.8 ± 0.9‰, *n* = 4). This leads us to suspect that the δ^13^C_SP_ values in the Betsiboka underestimate C_4_ vegetation cover, likely due to differences in pixel resolution between the Still and Powell (2010) output and our δ^13^C_SBE_ technique. Based on our δ^13^C_LITTER_ end-members in the Betsiboka basin, δ^13^C_SBE_ values estimate C_4_ plants may account for up to 96% of land cover within some sub-basins. Consideration of implicit errors of this method is briefly discussed in the Supplementary Materials.

### Bulk Riverine Carbon Concentrations and Carbon Stable Isotope Compositions

As could be expected, given the swath of grasslands covered by *lavakas* and the resultant sediment entrainment from these erosional features, streams and rivers in the Betsiboka basin were seasonally more sediment-laden than streams and rivers within the more heavily forested Rianila basin. Bulk TSM concentrations in the Betsiboka were significantly higher (SRT: *P* < 0.001, *n* = 28) during the wet season (range 2.1–3,589.5 mg l^−1^, average 464.0 ± 769.3 mg l^−1^, *n* = 29) than during the dry season (range 0.8–128.1 mg l^−1^, average 36.8 ± 40.3 mg l^−1^, *n* = 38), and whilst less variability occurred between seasons in the Rianila basin than the Betsiboka, bulk TSM concentration was also significantly elevated (SRT: *P* = 0.011, *n* = 16) during the wet season (range 3.6–103.2 mg l^−1^, average 32.4 ± 32.9 mg l^−1^, *n* = 16) than during the dry season (range 2.0–31.8 mg l^−1^, average 14.5 ± 9.0 mg l^−1^, *n* = 16).

**Figure 5:**
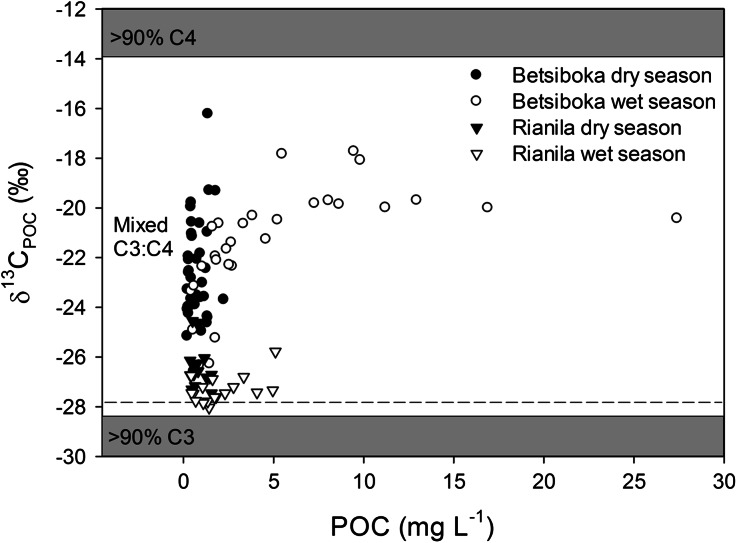
Carbon stable isotope signatures of riverine POC (δ^13^C_POC_) as a function of POC concentrations in the Betsiboka and the Rianila basins. *Grey shading* represent bounds for 90% C_3_ (δ^13^C_LITTER_ = −28.4‰) vegetation in the Rianila basin and 90% C_4_ (δ^13^C_LITTER_ = −13.9‰) vegetation for both the Rianila and Betsiboka basins, whereas the *horizontal dashed line* represents 90% C_3_ (δ^13^C_LITTER_ = −27.8‰) vegetation in the Betsiboka basin.

**Figure 6:**
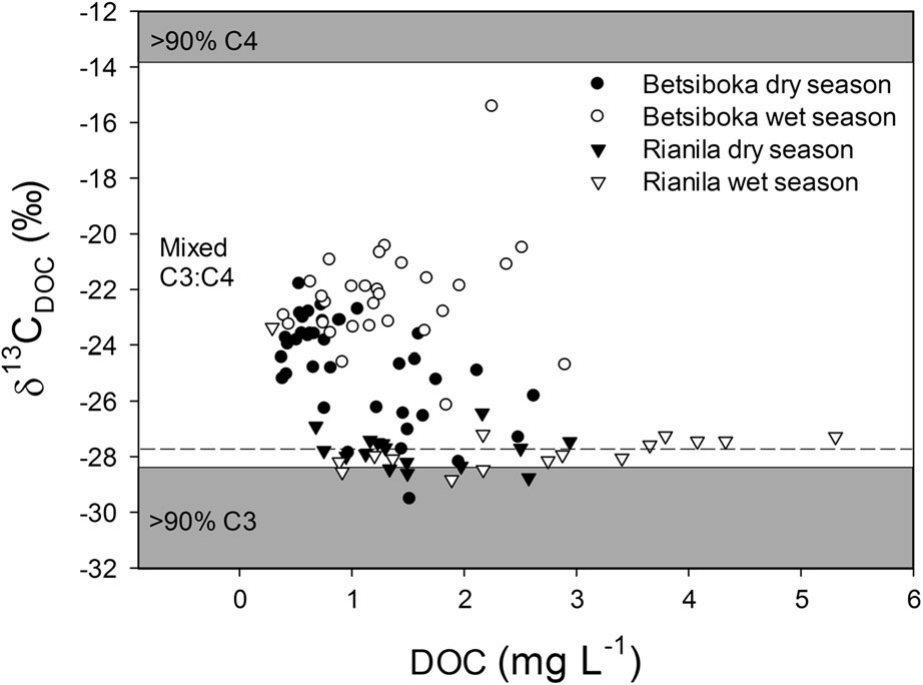
Carbon stable isotope signatures of riverine DOC (δ^13^C_DOC_) as a function of DOC concentrations in the Betsiboka and the Rianila basins. *Grey shading* represent bounds for 90% C_3_ (δ^13^C_LITTER_ = −28.4‰) vegetation in the Rianila basin and 90% C_4_ (δ^13^C_LITTER_ = −13.9‰) vegetation for both the Rianila and Betsiboka basins, whereas the *horizontal dashed line* represents 90% C_3_ (δ^13^C_LITTER_ = −27.8‰) vegetation in the Betsiboka basin.

Bulk riverine POC in the Betsiboka was significantly higher (SRT: *P* < 0.001, *n* = 28) during the wet season (range 0.4–27.4 mg C l^−1^, average 5.5 ± 6.0 mg C l^−1^, *n* = 29) than the dry season (range 0.2–2.2 mg l^−1^, average 0.8 ± 0.5 mg l^−1^, *n* = 38), and was represented by δ^13^C ranges of −26.3 to −17.7 and −26.6 to −16.2‰, respectively (Figure 5). The slight, yet significant (*P* = 0.001, *n* = 28), difference in δ^13^C_POC_ between dry season (−22.7 ± 2.0‰, *n* = 38) and wet season (−21.4 ± 2.3‰, *n* = 29) suggests that an increasing quantity of riverine OC is sourced from C_4_ grasslands during the wet season. Likewise, increased contribution from these landscape units would account for the marginally, yet again significantly, elevated DOC concentrations (Pt–T: *P* = 0.002, *n* = 28) and δ^13^C signatures (SRT: *P* < 0.001, *n* = 28) observed during the wet season (bulk DOC range 0.4–2.9 mg C l^−1^, average 1.3 ± 0.6 mg C l^−1^; δ^13^C_DOC_ range −26.2 to −15.4‰, average δ^13^C_DOC_ −22.3 ± 1.9‰; *n* = 29) relative to dry season (bulk DOC range 0.4–2.6 mg C l^−1^, average 1.1 ± 0.6 mg C l^−1^; δ^13^C_DOC_ range −29.5 to −21.8‰, average δ^13^C_DOC_ −24.8 ± 1.8‰, *n* = 38) (Figure 6).

Significantly elevated concentrations of POC (Pt–T: *P* = 0.014, *n* = 16) and DOC (Pt–T: *P* = 0.018, *n* = 16) were also observed in the streams and rivers of the Rianila basin during the wet season (POC: range 0.4–5.1 mg C l^−1^, average 2.1 ± 1.6 mg C l^−1^, *n* = 16; DOC: range 0.3–5.3 mg C l^−1^, average 2.6 ± 1.4 mg C l^−1^, *n* = 16) compared to the dry season (POC: range 0.4–1.8 mg C l^−1^, average 0.9 ± 0.5 mg C l^−1^, *n* = 16; DOC: range 0.7–2.9 mg C l^−1^, average 1.6 ± 0.7 mg C l^−1^, *n* = 16) (Figures 5, 6). Contrary to the enrichment observed in the Betsiboka basin, and evident from the dominance of C_3_ vegetation within the Rianila basin, riverine POC was significantly ^13^C-depleted (Pt–T: *P* = 0.002, *n* = 16) during the wet season (range −28.0 to −25.8‰, average −27.3 ± 0.6‰, *n* = 16) relative to dry season POC (range −27.6 to −24.6‰, average −26.5 ± 0.9‰, *n* = 16) in the Rianila basin. We speculate this may be attributed to an increased contribution of POC sourced from the wider Rianila basin (that is, less disturbed C_3_ forest), where there is less influence of human settlement (for example, logging of forest, agriculture, roads) on basin vegetation distribution. No significant seasonal shift in riverine DOC origin was found in the Rianila basin (wet season δ^13^C_DOC_ range −28.8 to −23.4‰, average −27.6 ± 1.2‰, *n* = 16; dry season δ^13^C_DOC_ range −28.8 to −26.4‰, average −27.8 ± 0.6‰, *n* = 16).

### Indicators of In-Situ Aquatic Production

Pelagic primary production in the Betsiboka basin during the dry season was generally negligible, ranging between 0.01 and 9.42 µmol C l^−1^ h^−1^ (average = 0.52 ± 1.54 µmol C l^−1^ h^−1^, *n* = 38), with all but three sites recording PPP rates below 1.0 µmol C l^−1^ h^−1^. Wet season PPP rates were not significantly different, ranging between 0.01 and 1.45 µmol C l^−1^ h^−1^ (average = 0.35 ± 0.35 µmol C l^−1^ h^−1^, *n* = 29). Despite low PPP rates in the Rianila across both seasons, PPP increased significantly (Pt–T: *P* ≤ 0.001, *n* = 14) during the wet season, with a range between 0.01 and 0.22 C l^−1^ h^−1^ (average = 0.07 ± 0.06 µmol C l^−1^ h^−1^, *n* = 15) during the dry season and 0.07 to 0.45 C l^−1^ h^−1^ (average = 0.20 ± 0.12 µmol C l^−1^ h^−1^, *n* = 15) in the wet season.

Dry season mg POC: mg Chl*a* ranged from 173 to 6,843 (average = 2,019 ± 1,241) and 1,280 to 4,468 (average = 2,768 ± 963) in the Betsiboka and Rianila basins, respectively. All wet season Chl*a* measurements from the Betsiboka basin were below the limit of quantification, which resulted in minimum values for mg POC: mg Chl*a* consistently above 1,000, except at B38 where the mg POC: mg Chl*a* was 487.

## Discussion

### Dominance of Terrestrial Organic Carbon

The major terrestrial OC sources considered here are C_3_ and C_4_ vegetation, though it is necessary to account for or dismiss the contribution from autochthonous sources, including phytoplankton, benthic algae and aquatic macrophytes, which in certain conditions can contribute substantially to riverine OC pools in tropical systems (Lewis and others 2001; Davies and others 2008). Several lines of evidence indicate that terrestrial carbon sources dominate in the basins studied here, and that any contributions from aquatic producers are minimal and would not bias our interpretations. First, the PPP rates were extremely low and showed little seasonal variation. Only downstream from the junction of the Ikopa with the Betsiboka, where channel slope rapidly decreases and the channel broadens, does PPP marginally increase (2.1–9.4 µmol C l^−1^ h^−1^). Second, low POC:Chl*a* ratios (~40; see Abril and others 2002, and references therein) are indicative of large phytoplankton contributions to the POC pool, with increasingly larger ratios where terrestrial-derived POC becomes more dominant (Finlay and Kendall 2007; Findlay 2010). Our data suggest negligible contribution of Chl*a* to riverine POC, with mg POC:mg Chl*a* consistently above 1,000 in both basins, and only once was the mg POC:mg Chl*a* less than 200 (in the main channel of the lower Betsiboka during dry season). Third, the Malagasy streams and rivers here were generally marked by low concentrations of ammonium (NH_4_^+^), nitrate (NO_3_^−^) and phosphate (PO_4_^3−^) across the two campaigns (Rianila median values: NH_4_^+^ = 6.3 µmol l^−1^, NO_3_^−^ = 3.0 µmol l^−1^, PO_4_^3−^ = 0.6 µmol l^−1^; Betsiboka median values: NH_4_^+^ = 4.1 µmol l^−1^, NO_3_^−^ = 2.7 µmol l^−1^, PO_4_^3−^ = 0.8 µmol l^−1^; data not presented in detail; see also Figure 9 of Marwick and others 2014).

The absence of substantial aquatic primary production can likely be ascribed to low nutrient and light conditions, through shading, for example, in the forested headwaters of the Rianila, or due to the high turbidity in the Betsiboka basin. Shading by riparian fringe vegetation has long been identified as an over-riding control on instream primary productivity (Feminella and others 1989; Boston and Hill 1991), and more recently in freshwaters of the tropics specifically (Davies and others 2008), whereas increased turbidity and light attenuation within the water column of larger rivers can also limit instream primary production (Vannote and others 1980). We speculate the latter may also be applicable to the grassland headwaters of the Betsiboka basin, which receive significant suspended sediment inputs from *lavakas*. Additionally, it has also been shown that, even where riparian shading effects are negligible, nutrients may impart substantial influence on instream primary productivity (Peterson and others 1983; Pringle and others 1986; Winterbourn 1990; Hill and others 1995; Borchardt 1996; Mosisch and others 1999), with evidence for nitrogen being the primary limiting nutrient for algal growth previously found in subtropical (Mosisch and others 2001) and tropical freshwaters (Downing and others 1999; Flecker and others 2002; Udy and Dennison 2005). No aquatic macrophytes were observed during the study, an observation consistent with Elouard (2001) who reports few macrophytes for Malagasy freshwaters as a whole. From the above reasoning, we speculate there is negligible contribution of autochthonous C to riverine OC pools of these Malagasy basins, and hypothesise that δ^13^C signatures of riverine particulate and DOC will be largely representative of the relative proportion of C originating from the two terrestrial end-members, that is, C_3_ and C_4_ vegetation.

### Disproportionate Contribution of Riparian OM to Riverine OC Pools

**Figure 7:**
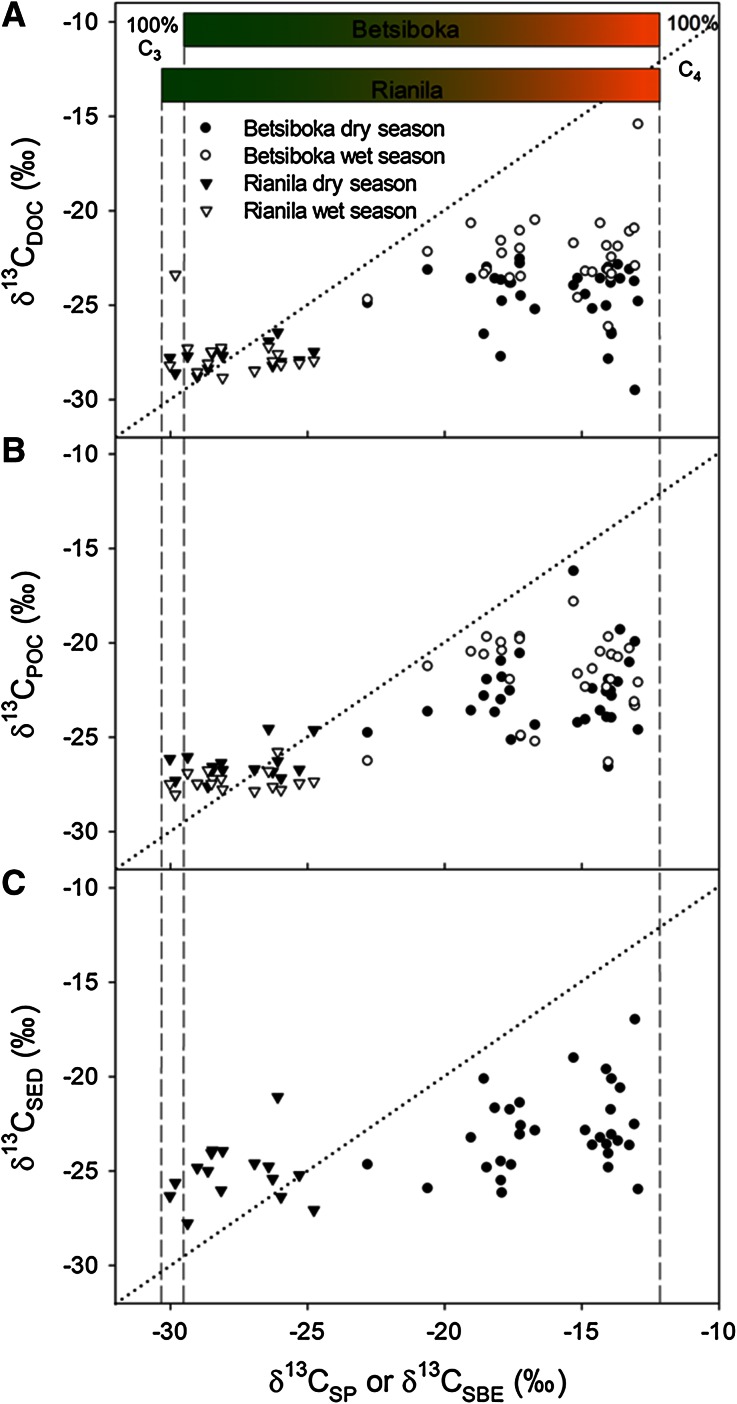
δ^13^C data on major riverine C pools (**A** DOC; **B** POC; **C** river sediment) along the gradient of sub-basin bulk vegetation δ^13^C (δ^13^C_SP_ or δ^13^C_SBE_). Sediment samples were only gathered during the dry season campaign. The *dotted line* represents a 1:1 relationship. *Vertical dashed lines* represent 100% C_3_ vegetation in the Rianila (δ^13^C_LITTER_ = −30.2‰) and Betsiboka (δ^13^C_LITTER_ = −29.5‰) and pure C_4_ (δ^13^C_LITTER_ = −12.2‰) vegetation in both basins.

**Figure 8:**
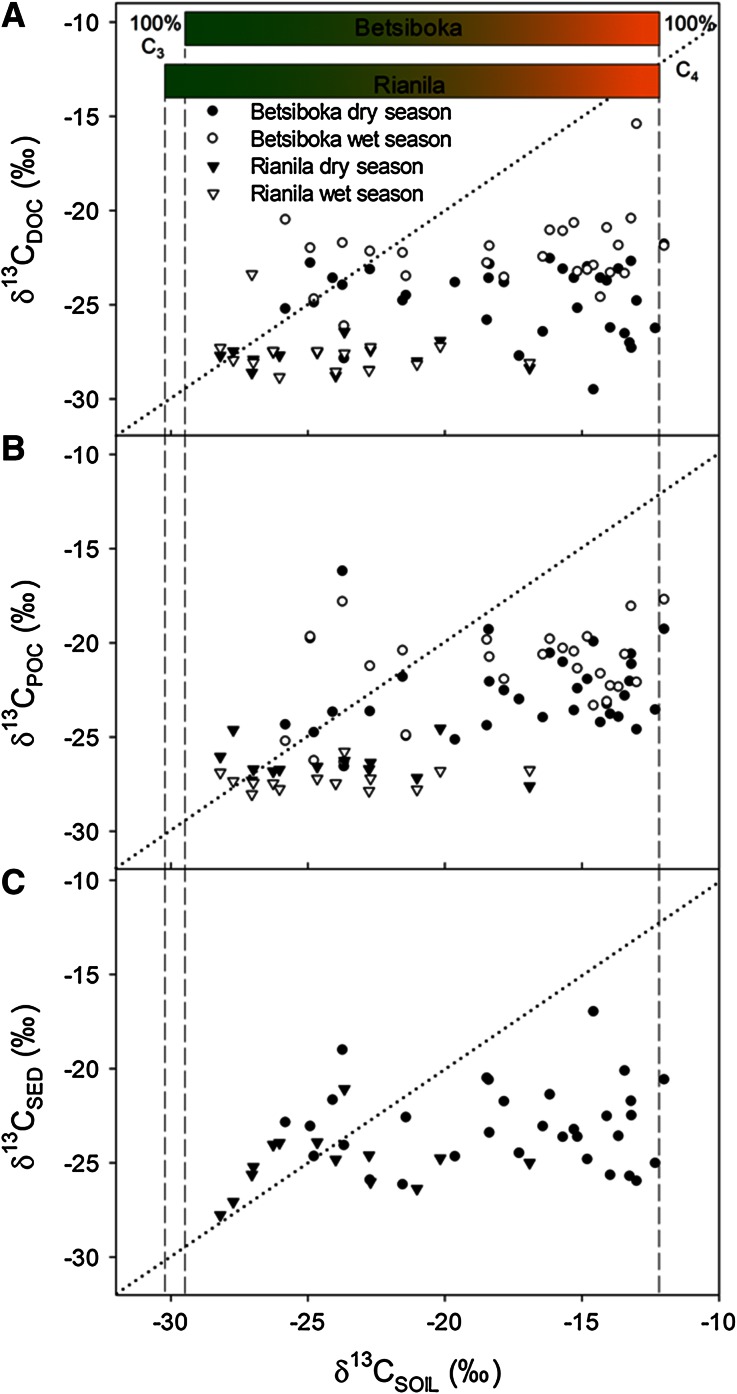
δ^13^C data on major riverine C pools (**A** DOC; **B** POC; **C** river sediment) along the gradient of sub-basin soil organic carbon δ^13^C (δ^13^C_SOIL_). Soil and sediment samples were only gathered during the dry season campaign. The *dotted line* represents a 1:1 relationship. *Vertical dashed lines* represent 100% C_3_ vegetation in the Rianila (δ^13^C_LITTER_ = −30.2‰) and Betsiboka (δ^13^C_LITTER_ = −29.5‰) and pure C_4_ (δ^13^C_LITTER_ = −12.2‰) vegetation in both basins.

A clear pattern emerges when considering the combined data from both basins, with riverine DOC, POC and sediment OC all consistently more ^13^C-depleted than expected across the C_3_–C_4_ gradient. These relationships are evident irrespective of whether riverine OC fractions are explored as a function of estimated bulk vegetation δ^13^C (Figure 7A–C) or soil δ^13^C (Figure 8A–C).

Excluding lesser autochthonous contributions, riverine DOC is sourced from the leaching of plant litter and the degradation of soil OM, whereas the POC fraction, on the other hand, is largely sourced from fragmented plant matter and direct inputs of leaf litter, OM mobilised from surrounding soil profiles, as well as autotrophic production within the freshwaters (Meybeck 1993; Ludwig and others 1996; Finlay and Kendall 2007). Having already discounted significant contribution of algal C sources to riverine OC pools in the Rianila basin, it is unsurprising that riverine DOC and POC δ^13^C values reflect the dominance of C_3_ vegetation in this system (Figure 7A, B). Additionally, recent radiocarbon measurements of DOC in the Rianila basin during the wet season, including two headwater and one main channel samples, were all ^14^C-enriched (∆^14^C range +81 to +136‰; own unpublished data) and indicate a modern terrestrial C_3_-enriched DOC source for Rianila streams and rivers.

The relationship between soils and riverine OC in the Rianila basin (Figure 8A, B) is weaker than that discussed above for basin vegetation, though can be expected given that riverine δ^13^C values and δ^13^C_VEG_ estimates are integrated compositions of δ^13^C from broad areas, whereas δ^13^C_SOIL_ values are determined from spatially isolated samples. Although δ^13^C_SOIL_ values do highlight the, at times significant (up to −16.9‰), presence of C_4_ biomass within the vicinity of Rianila streams and rivers. We speculate that the presence of this C_4_ biomass within the riparian fringe and surrounding floodplain, likely associated with the tendency of human settlement and disturbance of the natural vegetation within these areas (including the introduction of C_4_ crops and invasive species), results in the slight ^13^C enrichment observed in the riverine POC pool during the dry season. Others have shown that the riparian fringe disproportionately contributes to dry season riverine POC loads in the tropics (Mariotti and others 1991; Bird and others 1992). In the case of the Rianila, elevated rainfall would lead to increased quantities of OM mobilised from the C_3_-enriched surrounding forests, subsequently lowering the relatively ^13^C-enriched dry season POC signal.

Considering δ^13^C_VEG_ estimates suggest that C_4_ biomass accounts for between 39 and 96% of vegetation cover in the Betsiboka sub-basins, there is no such prominence of C_4_ derived C in riverine OC pools (Figure 7A, B). A similar observation has been made for other subtropical and tropical savannah/grassland ecosystems (Mariotti and others 1991; Bird and others 1992, 1998; Bird and Pousai 1997). For example, Mariotti and others (1991) report an average riverine δ^13^C_POC_ value of −27.5‰ for three savannah dominated tributaries of the Congo River, whereas Bird and others (1992) provide values ranging from −29.9 to −27.0‰ for the cerrado-dominated Araguaia/Tocantins sub-basin of the Amazon network. A seasonal fluctuation in riverine δ^13^C_POC_ of 3.3‰ was also identified in the Sanaga River basin by Bird and others (1998) and closely linked to runoff intensity, with the lower δ^13^C values present during the wet season, which is comparable to the average deviation of 1.1‰ in streams and rivers of the Betsiboka where we have both dry and wet season δ^13^C_POC_ data (*n* = 28). A common feature to each of these basins for which δ^13^C_POC_ values are listed above, including the Betsiboka basin here, is the C_3_-enriched nature of the riparian fringes. It is generally considered, and re-iterated here through our observations in the Betsiboka basin, that the C_3_-enriched riparian fringe, being in close proximity to the riverbanks, provides disproportionately (relative to land cover) to dry season riverine OC pools, whereas with the advent of the wet season, the increased mobilisation of OC from the C_4_-enriched areas farther from the streams and rivers leads to relatively ^13^C-enriched riverine POC pools (Mariotti and others 1991; Bird and others 1992, 1998; Bird and Pousai 1997).

Although we consider the disconnection of the wider C_4_-dominated basin from the streams and rivers as the primary driver of the under-representation of C_4_-derived C within riverine OC pools in the Betsiboka, given the history (Burney 1987a, b, c) and prevalence (Kull 2004, 2012) of fire within the grasslands of Madagascar, the effect of terrestrial biomass loss through combustion by fire needs some consideration. Kull (2004) reports as much as one quarter to one half (of land area) of the Malagasy grasslands may be burned annually. Lehsten and others (2009) estimate African wildfires consume approximately 10% of savannah NPP annually, approximating 0.52–0.59 t C ha^−1^ y^−1^ from Lloyd et al. (2008) estimates of African grassland NPP (see discussion below). Clearly, the preferential loss of C_4_ derived C to the atmosphere as CO_2_ or fine particulate matter by combustion diminishes the quantity available for export to streams and rivers (Menaut and others 1990) and thereby should be considered a subsidiary control of riverine OC δ^13^C composition.

Where only a single sampling of riverine OC is logistically possible in tropical basins containing significant C_4_ biomass, due to the large annual range of riverine δ^13^C_POC_ values, the δ^13^C values of river bed sediments offer a more reliable indication of the average riverine δ^13^C_POC_ transported in these systems (Bird and Pousai 1997). Although here we replicate riverine OC measurements in the dry and wet season, it is reasonable to assume our data do not capture the total annual variability of riverine POC and, as such, our river bed sediment δ^13^C values may be a more reliable indicator of average riverine δ^13^C_POC_.

The limited range of δ^13^C_SED_ values relative to δ^13^C_SOIL_ values (Figure 8C) in both the Rianila (average δ^13^C_SED_ −25.1 ± 1.5‰, *n* = 16) and Betsiboka (average δ^13^C_SED_ −23.0 ± 2.1‰, *n* = 37) is normal given that river bed sediments assimilate δ^13^C compositions from a considerably broader area relative to the soil measurements (Bird and Pousai 1997). Whereas δ^13^C_SED_ values in the Rianila basin were largely reflective of our δ^13^C_VEG_ estimates (Figure 7C), the significant depletion of ^13^C in river bed sediments of the Betsiboka basin compared to our δ^13^C_VEG_ estimates (average δ^13^C_SED_–δ^13^C_VEG_ 6.9 ± 2.7‰, *n* = 32) confirms the suggestions above that there exists a strong disconnection between the terrestrial C_4_ and riverine OC pools. Such a disconnection may lead to gross over-estimation of basin-scale C_3_ vegetation cover when extrapolated from δ^13^C signatures stored in sedimentary deposits of fluvial origin.

Two regional environmental conditions may complicate our interpretations outlined above. First, if net primary productivity (NPP) in C_3_-enriched riparian fringes is significantly greater relative to NPP of the surrounding grasslands, it may not be surprising to find an under-representation of C_4_ sourced C (relative to land cover) in riverine OC pools. We do not believe the riparian fringe vegetation in the Betsiboka basin constitutes tropical forests, an African biome for which no published field studies estimating NPP exist (Valentini and others 2014), but rather more closely resemble scrub and woodland savannah as defined by Torello-Raventos and others (2013). Employing the NPP estimates provided by Lloyd and others (2008), which cover the transition from grassland to woodland savannah in the tropics, grassland NPP may be between 5.2 and 5.9 t C ha^−1^ y^−1^, which is comparable with the median estimate of 6.2 ± 1.8 t C ha^−1^ y^−1^ reported by Ciais and others (2011). For open savannah woodland in eastern and southern Africa, Lloyd and others (2008) estimate NPP of 7.8 and 5.1 t C ha^−1^ y^−1^, respectively, and values of 8.8 and 7.1 t C ha^−1^ y^−1^ for eastern and southern savannah woodlands, respectively. It should also be noted that the riparian fringes of the Betsiboka basin are not spatially expansive, and where present often do not extend more than a few tens of metres from the river (see Figure 3; Supplementary Materials). As such, we suspect NPP in the grasslands and riparian fringe of the Betsiboka are within a comparable range, and any differences will have a minor impact on the quantity of terrestrial C available for export to riverine C pools from the respective landscape units. Second, C_4_ agricultural crops, such as sugar cane, may contribute significantly to riverine OC pools (Bunn and others 1997). The area of sugar cane harvest in both the Betsiboka and Rianila basins equates to less than 0.2% of total basin area (HarvestChoice 2011), and as no major sugar cane agriculture was observed within the vicinity of any of the field sites, we expect it to be a negligible source of C_4_ carbon to the riverine pools reported here. Whereas, in Malagasy basins paddy rice agriculture, a C_3_ crop, is often confined to the riparian fringe (Aldegheri 1964; Benstead and Pringle 2004), and could potentially skew riverine OC δ^13^C values towards the C_3_ end-member.

## Conclusions

With their strong partitioning of C_3_ and C_4_ vegetation at the landscape scale, subtropical and tropical C_4_-rich grasslands provide the optimum environment to further explore the relative delivery of terrestrial C from different landscape units to river networks, as well as the investigation of trophic linkages between aquatic consumers and riparian carbon sources (for example, Abrantes and others 2013). Our data on the δ^13^C signatures of various riverine OC pools from subtropical basins demonstrate that the C_3_-enriched riparian vegetation contributes disproportionately to riverine OC pools compared to the spatially dominant, yet seasonally disconnected, C_4_-dominant grasslands. The prevalence of fire within grassland ecosystems, with consideration they may combust 10% of annual NPP within this biome type, suggests the prevailing fire regime will also exert significant control over the quantity of grassland OM available for export to streams and rivers and, ergo, riverine OC δ^13^C values. These observations clearly highlight that the δ^13^C of riverine organic C pools may not be an accurate proxy for extrapolating C_3_ and C_4_ vegetation cover of a drainage basin, but appear to be strongly biased towards the dominant photosynthetic pathway of riparian fringe vegetation. Our δ^13^C values of riverine sediments in a basin dominated by tropical grassland indicate that upon deposition these sediments are not representative of the overall vegetation distribution in the contributing drainage basin, and that the analysis of sedimentary δ^13^C signatures for the purpose of paleo-reconstruction of vegetation distribution (Cerling and others 1988; Mora and others 2002; Santschi and others 2007; dos Santos and others 2013) may lead to significant over-estimation of the areal C_3_ cover within drainage basins characterised by considerable expanses of C_4_ grassland.

## Electronic supplementary material

Below is the link to the electronic supplementary material.

Supplementary material 1 (DOCX 97 kb)

Supplementary material 2 (TIFF 3439 kb)

Supplementary material 3 (TIFF 4656 kb)

Supplementary material 4 (TIFF 3513 kb)

Supplementary material 5 (TIFF 1969 kb)

Supplementary material 6 (TIFF 3169 kb)

Supplementary material 7 (TIFF 3072 kb)

Supplementary material 8 (TIFF 3226 kb)

Supplementary material 9 (TIFF 3182 kb)

Supplementary material 10 (TIFF 2970 kb)

Supplementary material 11 (TIFF 3673 kb)

Supplementary material 12 (TIFF 3053 kb)
