# Supplementary material for: Disproportionate Contribution of Riparian Inputs to Organic Carbon Pools in Freshwater Systems
Source: Ecosystems. 2014 Apr 29;17(6):974–89. doi: 10.1007/s10021-014-9772-6 (PMC4133959; doi:10.1007/s10021-014-9772-6)
Supplement: Supplementary file 1 — Supplementary material 1 (DOCX 97 kb) [file 10021_2014_9772_MOESM1_ESM.docx]

**Disproportionate contribution of riparian inputs to organic carbon pools in freshwater systems.**

Trent R. Marwick^1^* ([trentrichard.marwick@ees.kuleuven.be](mailto:trentrichard.marwick@ees.kuleuven.be)) Ph: +32 16 372 228

Alberto Vieira Borges^2^ ([alberto.borges@ulg.ac.be](mailto:alberto.borges@ulg.ac.be))

Kristof Van Acker^1^ ([vanacker.kristof@gmail.com](mailto:vanacker.kristof@gmail.com))

François Darchambeau^2^ ([francois.darchambeau@ulg.ac.be](mailto:francois.darchambeau@ulg.ac.be))

Steven Bouillon^1^ ([steven.bouillon@ees.kuleuven.be](mailto:steven.bouillon@ees.kuleuven.be))

***** *Corresponding author.*

*^1^Katholieke Universiteit Leuven (KU Leuven), Department of Earth and Environmental Sciences, Celestijnenlaan 200E, B-3001 Leuven, Belgium. ^2^Unité d’Océanographie Chimique, Université de Liège (ULg), Belgium.*

**Supplementary Material**

The following file provides (i) the raw data presented in the main text, and (ii) further examples of our technique for estimating C_3_ and C_4_ vegetation distribution in the Betsiboka basin (δ^13^C_SBE_).

We believe our δ^13^C_SBE_ estimates more accurately reflect C_3_ and C_4_ distribution on a local scale in the Betsiboka basin than estimates derived from Still and Powell (2010), although some error is implicit with our method. For example, the Still and Powell (2010) model accounts for the contribution of agricultural crops, whereas no crop correction is performed through the δ^13^C_SBE_ procedure. Yet, floodplains in the Betsiboka basin are often engineered for rice (C_3_) production (Aldegheri 1964), the dominant crop and staple diet of much of the Malagasy population. Also, the presence of clouds, bare ground, and other shadow-related effects all contribute to error in the classification of pixels. The influence of clouds and shadow-related effects are largely avoided by selective placement of the representative vegetation quadrants used to calculate δ^13^C_SBE_ (*see* Supplementary Material), though we expect the presence of bare-ground, interspersed by grass tussocks, may to varying extents contribute to the C_4_ end-member. As such, we posit that actual Betsiboka vegetation δ^13^C will likely be within the range of δ^13^C_SBE_ and δ^13^C_SP_ estimates.

**References**

Aldegheri M. 1964. Monographie hydrologique de l’Ikopa et de la Betsiboka: Facteurs conditionnels du régime. Paris: ORSTOM. 76p.

Still CJ, Powell RL. 2010. Continental-scale distributions of vegetation stable carbon isotope ratios. West JB, Bowen GJ, Dawson TE, Tu KP, editors. Isoscapes. Netherlands: Springer Netherlands. p179–193.

1. **Raw data**

Continued next page

Continued next page

1. **δ^13^C_SBE_ estimate examples**

Supplementary Figure 1. Overview of sub-basins (green polygons) where δ^13^C_SBE_ values were estimated in the Betsiboka basin (larger white polygon). The Mahajamba sub-basin, an intermittent tributary to the Betsiboka, is bounded by the smaller white polygon situated to the east of the Betsiboka basin. A total of 21 sub-basins were analysed, with an altitude range (at the sampling point) between 49 and 1644 metres above sea level. (Data sourced from Google Earth with data provided by SIO, NOAA, U.S. Navy, NGA and GEBCO)

Supplementary Figure 2. (a) Sub-basin boundary (green polygon) for site B3, showing the quadrants used to estimate δ^13^C_SBE_ (orange polygons). (b) Satellite image of the quadrants and (c) the quadrants converted to greyscale, where black represents C_3_ vegetation. Photograph of the sub-basin (d-e) with the field-of-view indicated by the red dot in (a). (Data sourced from Google Earth with data provided by DigitalGlobe and Cnes/Spot Image)

Supplementary Figure 3. (a) Sub-basin boundary (green polygon) for site B17, showing the quadrants used to estimate δ^13^C_SBE_ (orange polygons). (b) Satellite image of the quadrants and (c) the quadrants converted to greyscale, where black represents C_3_ vegetation. Photograph of the sub-basin (d) with the field-of-view indicated by the red arrows in (a). (Data sourced from Google Earth with data provided by DigitalGlobe, Cnes/Spot Image and CNES/Astrium)

Supplementary Figure 4. (a) The sub-basin (green polygon) for site B20 which was used to calculate δ^13^C_SBE_. (b) Satellite image extract of the sub-basin and (c) the sub-basin converted to greyscale, where black represents C_3_ vegetation. The typical landscape segregation of C_3_ and C_4_ biomass in surrounding tributaries of Betsiboka River is displayed in (d) (photo taken in the valley adjacent to the B20 sub-basin, with the field-of-view indicated by the red arrows in (a)). (Data sourced from Google Earth with data provided by DigitalGlobe)

Supplementary Figure 5. (a) The sub-basin (green polygon) for site B21 which was used to calculate δ^13^C_SBE_. (b) Satellite image extract of the sub-basin and (c) the sub-basin converted to greyscale, where black represents C_3_ vegetation. Photograph of the sub-basin (d) highlighting the predominance of C_4_ biomass (the field-of-view is indicated by the red arrows in (a)). (Data sourced from Google Earth with data provided by DigitalGlobe and Cnes/Spot Image)

Supplementary Figure 6. (a) The sub-basin (green polygon) for site B23 which was used to calculate δ^13^C_SBE_. (b) Satellite image extract of the sub-basin and (c) the sub-basin converted to greyscale, where black represents C_3_ vegetation. Photograph of the *lavaka*-riddled sub-basin (d) during the wet season (the field-of-view is indicated by the red arrows in (a)). (Data sourced from Google Earth with data provided by DigitalGlobe and CNES/Astrium)

Supplementary Figure 7. (a) The sub-basin (green polygon) for site B24 which was used to calculate δ^13^C_SBE_. (b) Satellite image extract of the sub-basin and (c) the sub-basin converted to greyscale, where black represents C_3_ vegetation. Photograph of the sub-basin (d) (the field-of-view is indicated by the red arrows in (a)). (Data sourced from Google Earth with data provided by DigitalGlobe and CNES/Astrium)

Supplementary Figure 8. (a) The sub-basin (green polygon) for site B25 which was used to calculate δ^13^C_SBE_. (b) Satellite image extract of the sub-basin and (c) the sub-basin converted to greyscale, where black represents C_3_ vegetation. (Data sourced from Google Earth with data provided by DigitalGlobe and Cnes/Spot Image)

Supplementary Figure 9. (a) Sub-basin boundary (green polygon) for site B27, showing the quadrant (orange polygon) used to calculate δ^13^C_SBE_. (b) Satellite image of the quadrant and (c) the quadrant converted to greyscale, where black represents C_3_ vegetation. (Data sourced from Google Earth with data provided by DigitalGlobe and NASA)

Supplementary Figure 10. (a) The sub-basin (green polygon) for site B38 which was used to calculate δ^13^C_SBE_. (b) Satellite image extract of the sub-basin and (c) the sub-basin converted to greyscale, where black represents C_3_ vegetation. Photograph of the sub-basin (d) (the field-of-view is indicated by the red arrows in (a)). (Data sourced from Google Earth with data provided by DigitalGlobe)

Supplementary Figure 11. (a) Sub-basin boundary (green polygon) for site B40, showing the quadrant (orange polygon) used to calculate δ^13^C_SBE_. (b) Satellite image of the quadrant and (c) the quadrant converted to greyscale, where black represents C_3_ vegetation. Dry (d) and wet (e) season photographs of the region surrounding the sub-basin (photos located south of the B40 boundary, though they are representative of the conditions observed with the sub-basin). (Data sourced from Google Earth with data provided by DigitalGlobe)
