# Supplementary figures and images for: Disproportionate Contribution of Riparian Inputs to Organic Carbon Pools in Freshwater Systems
Source: Ecosystems. 2014 Apr 29;17(6):974–89. doi: 10.1007/s10021-014-9772-6 (PMC4133959; doi:10.1007/s10021-014-9772-6)

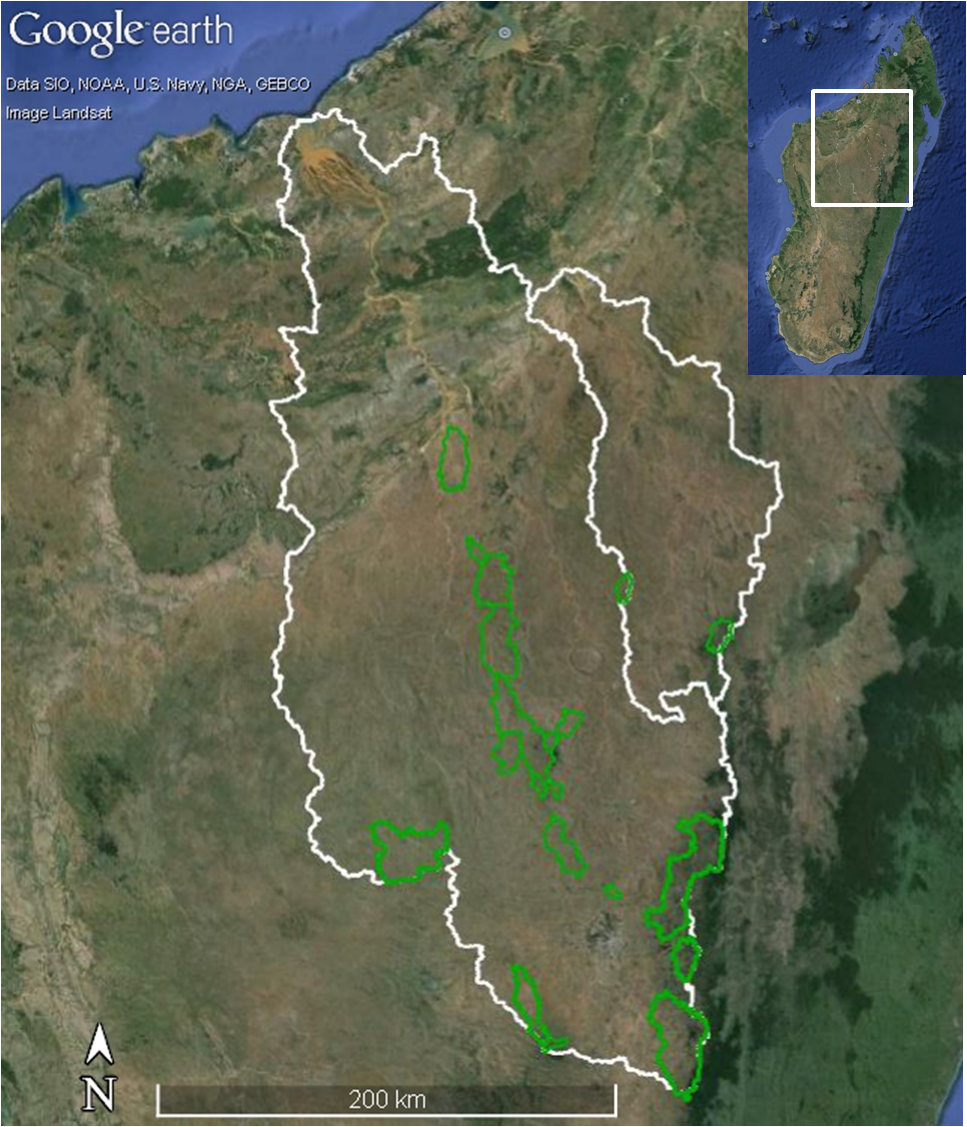

Supplement: Supplementary file 2 — Supplementary material 2 (TIFF 3439 kb) [file 10021_2014_9772_MOESM2_ESM.tiff]

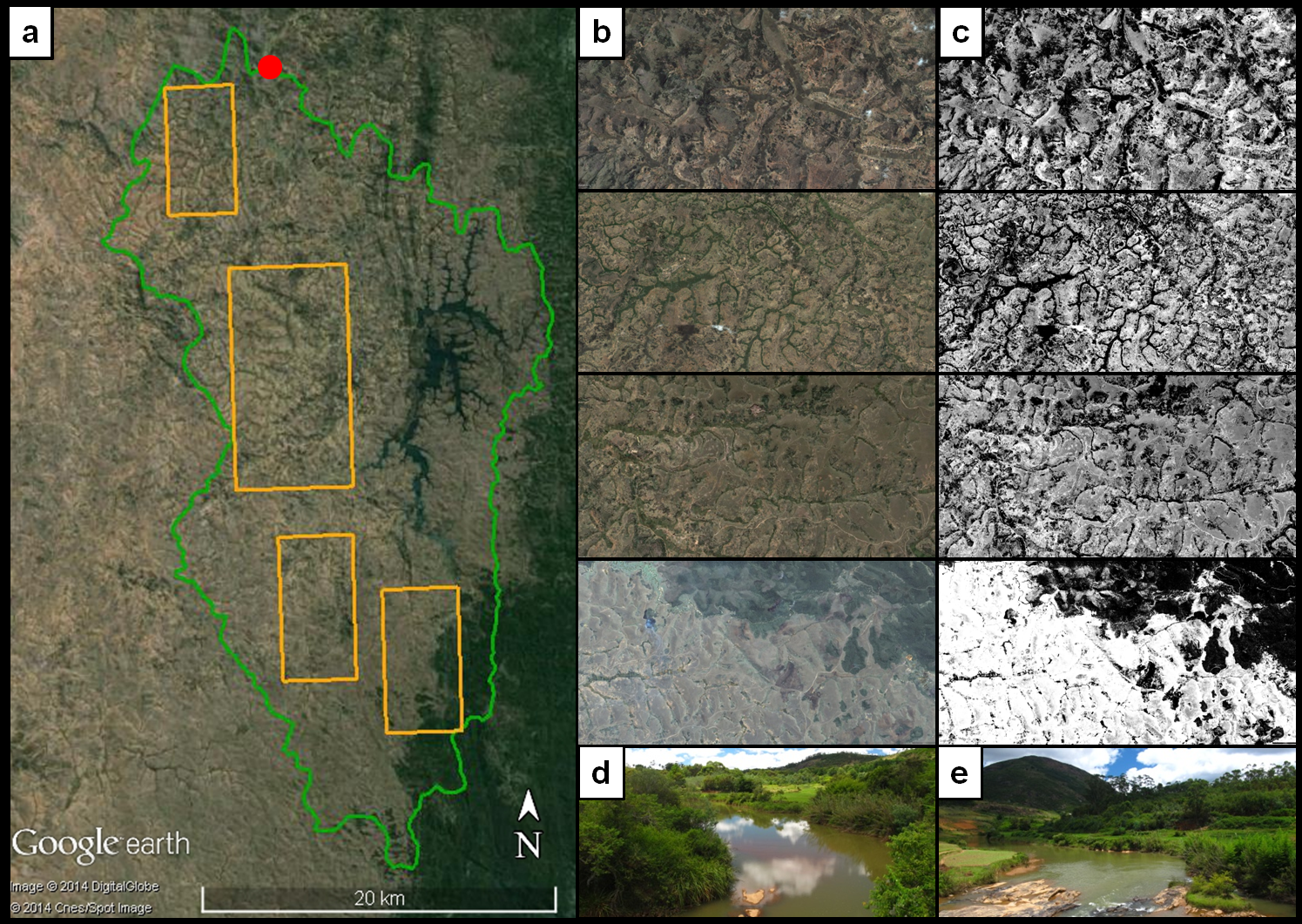

Supplement: Supplementary file 3 — Supplementary material 3 (TIFF 4656 kb) [file 10021_2014_9772_MOESM3_ESM.tiff]

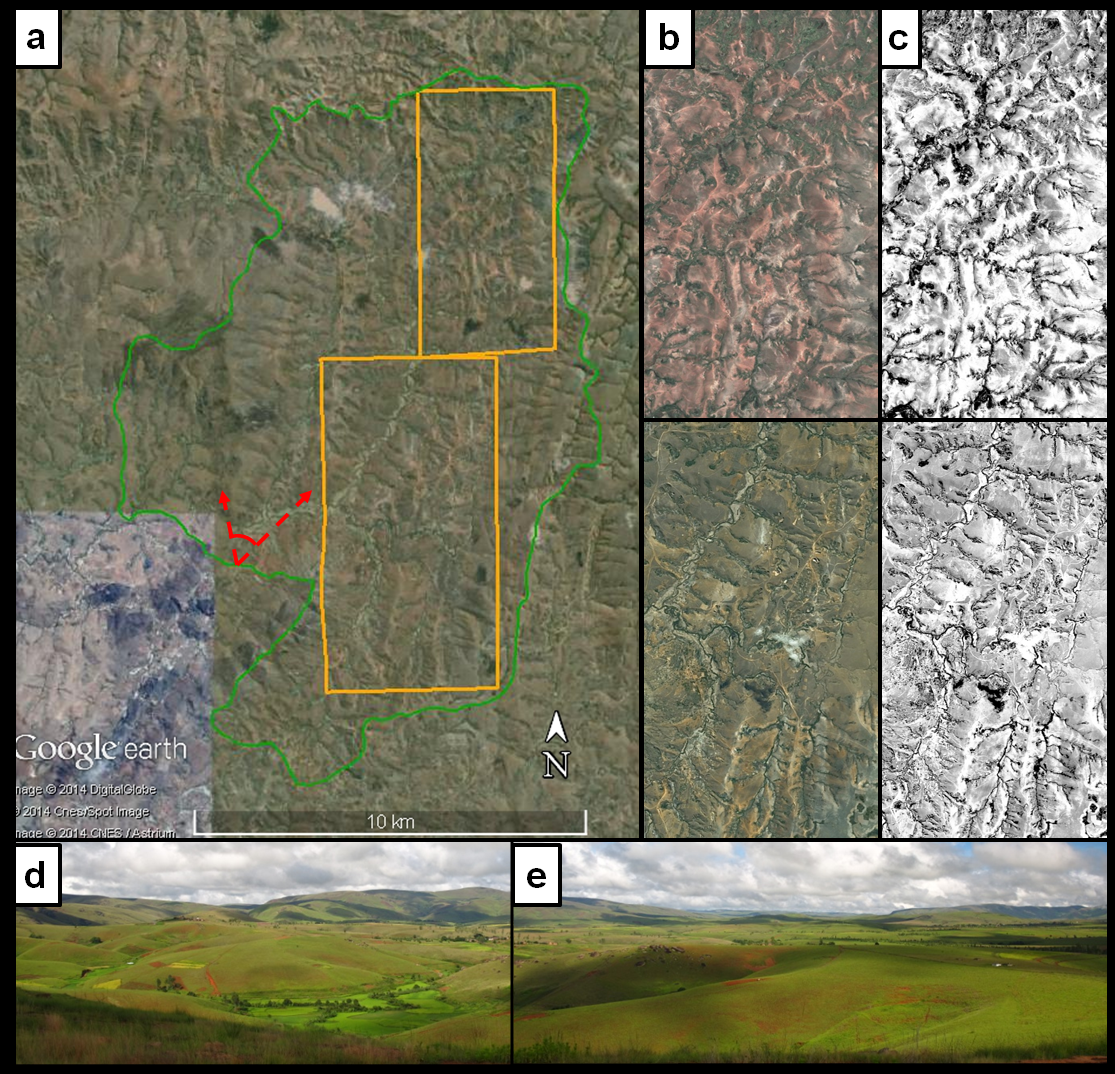

Supplement: Supplementary file 4 — Supplementary material 4 (TIFF 3513 kb) [file 10021_2014_9772_MOESM4_ESM.tiff]

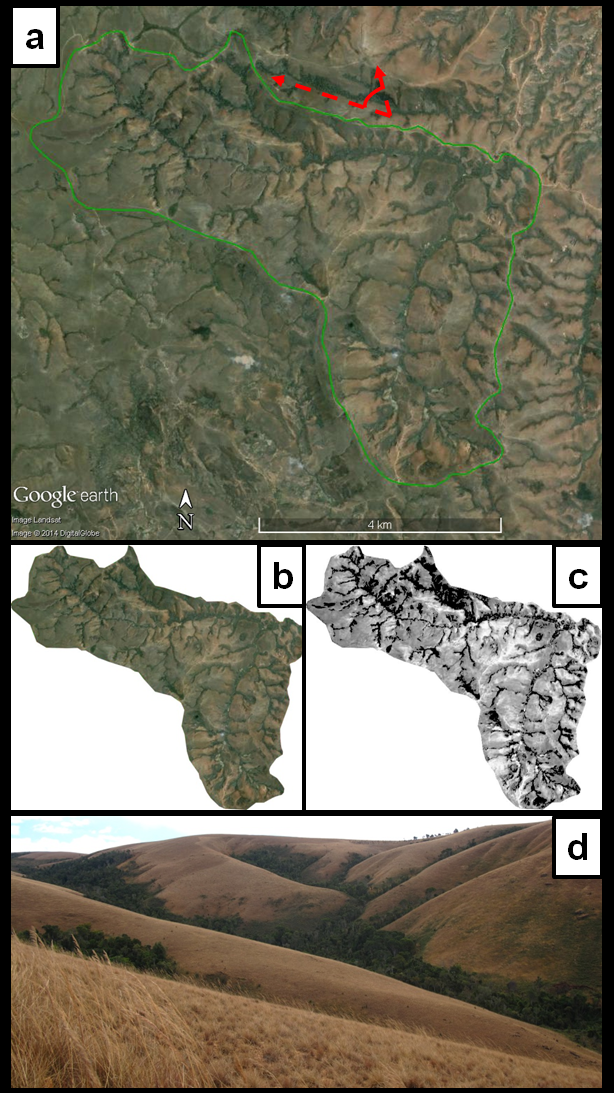

Supplement: Supplementary file 5 — Supplementary material 5 (TIFF 1969 kb) [file 10021_2014_9772_MOESM5_ESM.tiff]

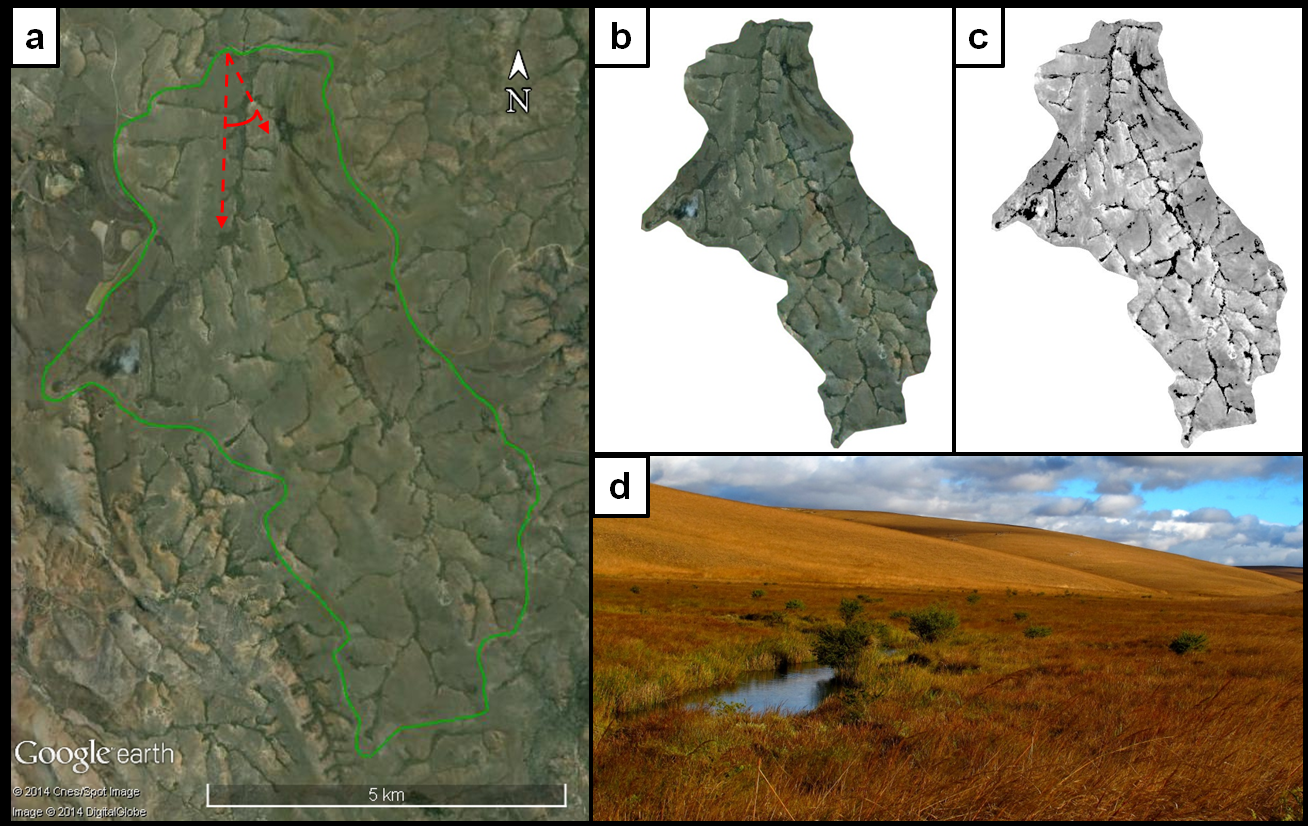

Supplement: Supplementary file 6 — Supplementary material 6 (TIFF 3169 kb) [file 10021_2014_9772_MOESM6_ESM.tiff]

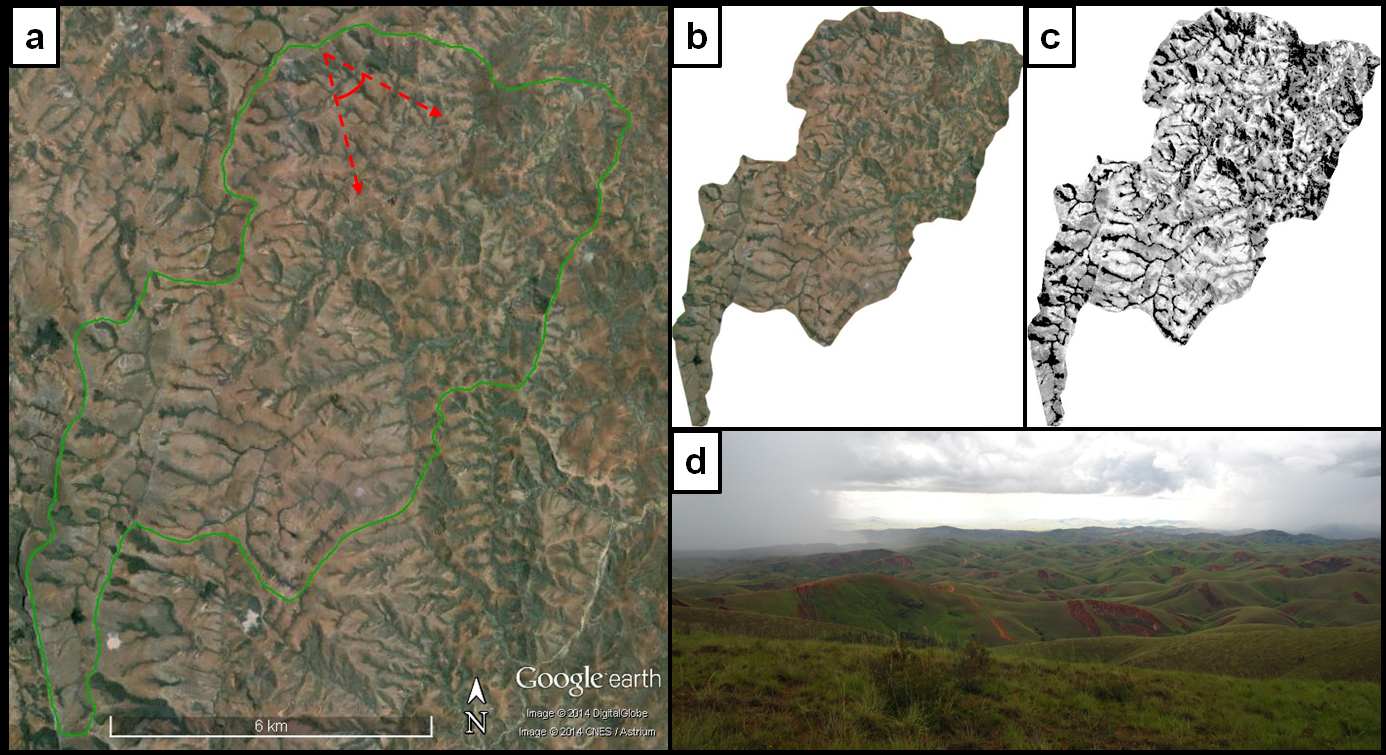

Supplement: Supplementary file 7 — Supplementary material 7 (TIFF 3072 kb) [file 10021_2014_9772_MOESM7_ESM.tiff]

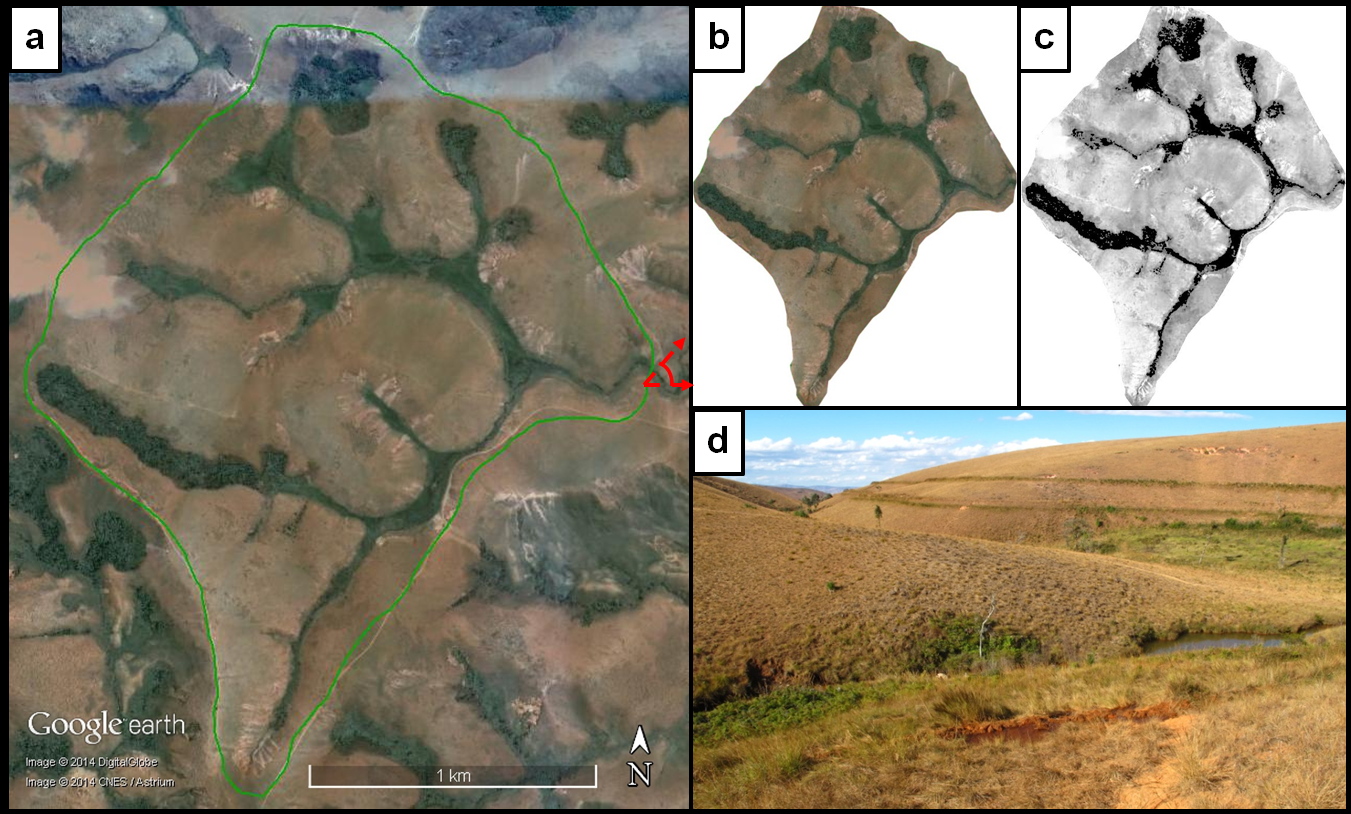

Supplement: Supplementary file 8 — Supplementary material 8 (TIFF 3226 kb) [file 10021_2014_9772_MOESM8_ESM.tiff]

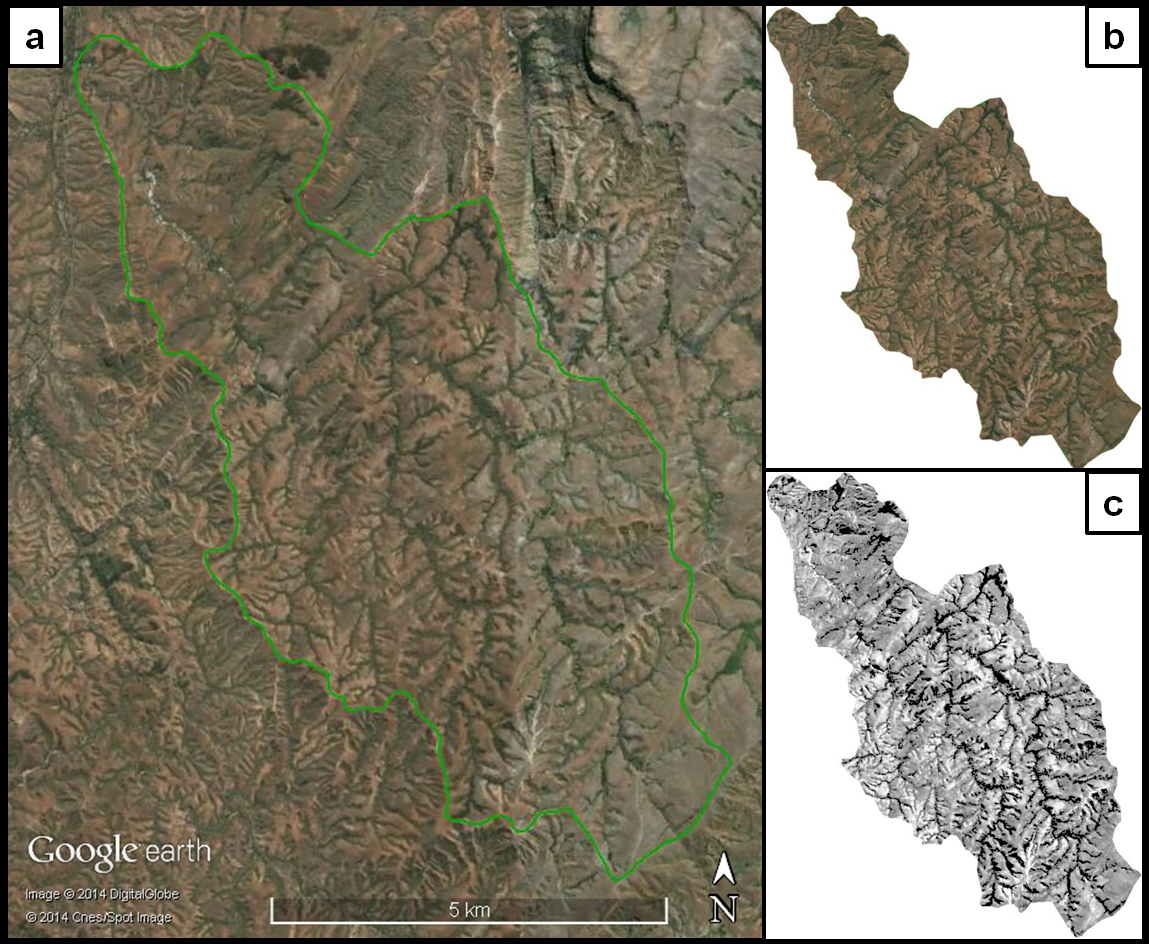

Supplement: Supplementary file 9 — Supplementary material 9 (TIFF 3182 kb) [file 10021_2014_9772_MOESM9_ESM.tiff]

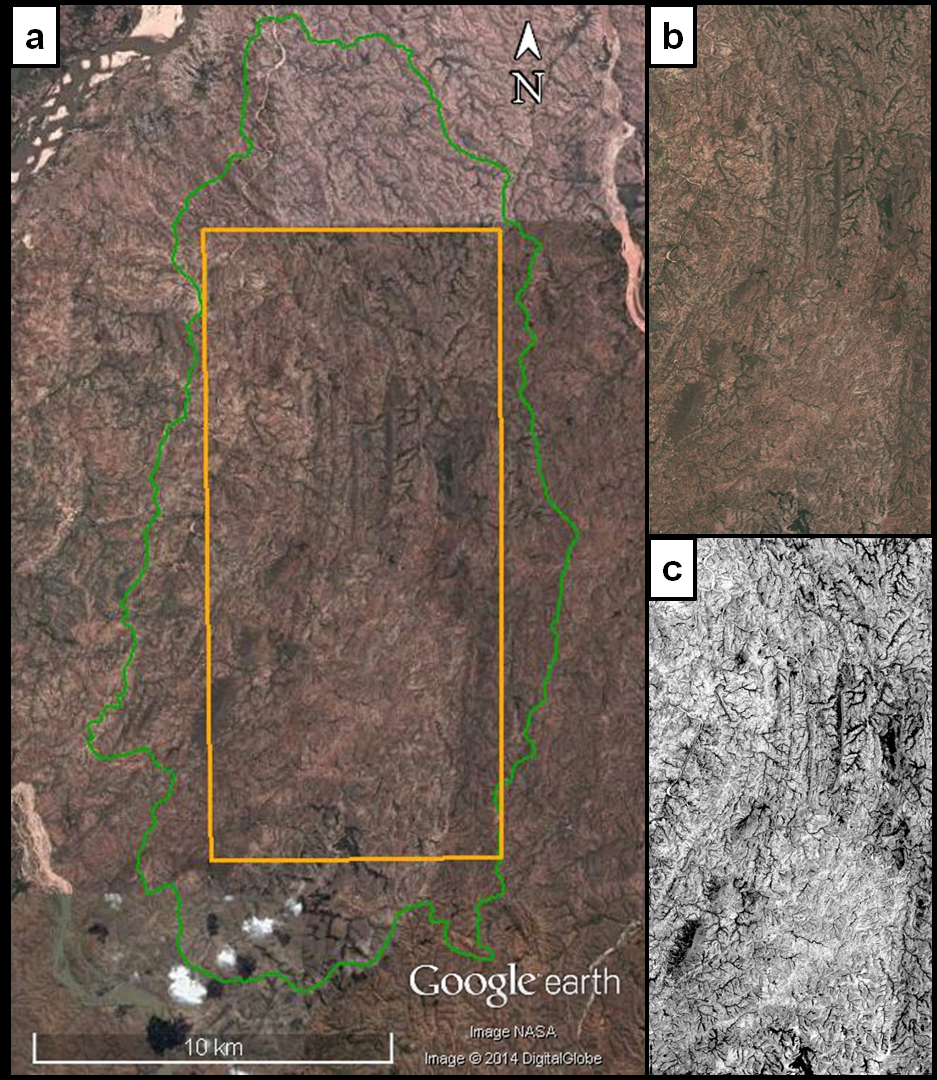

Supplement: Supplementary file 10 — Supplementary material 10 (TIFF 2970 kb) [file 10021_2014_9772_MOESM10_ESM.tiff]

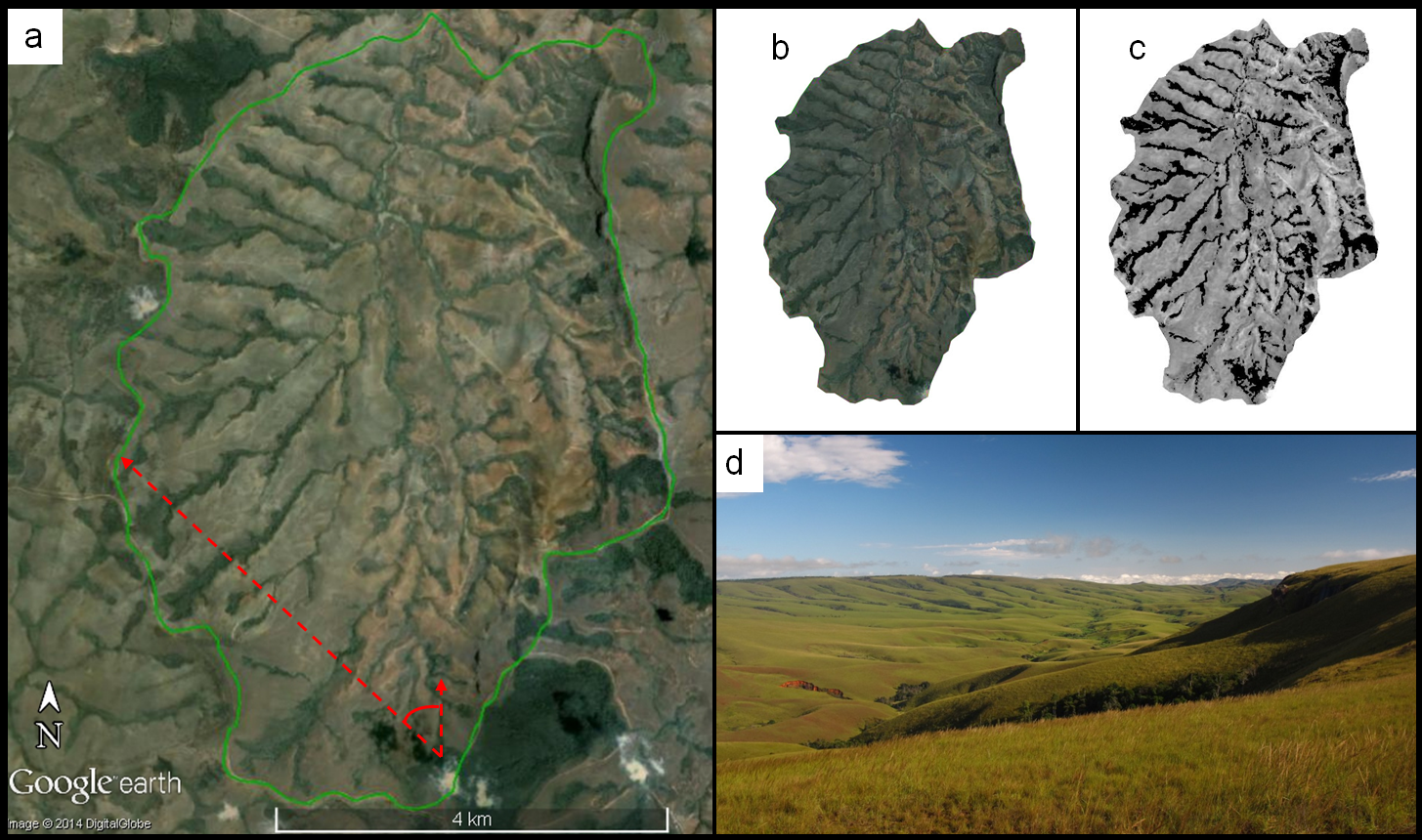

Supplement: Supplementary file 11 — Supplementary material 11 (TIFF 3673 kb) [file 10021_2014_9772_MOESM11_ESM.tiff]

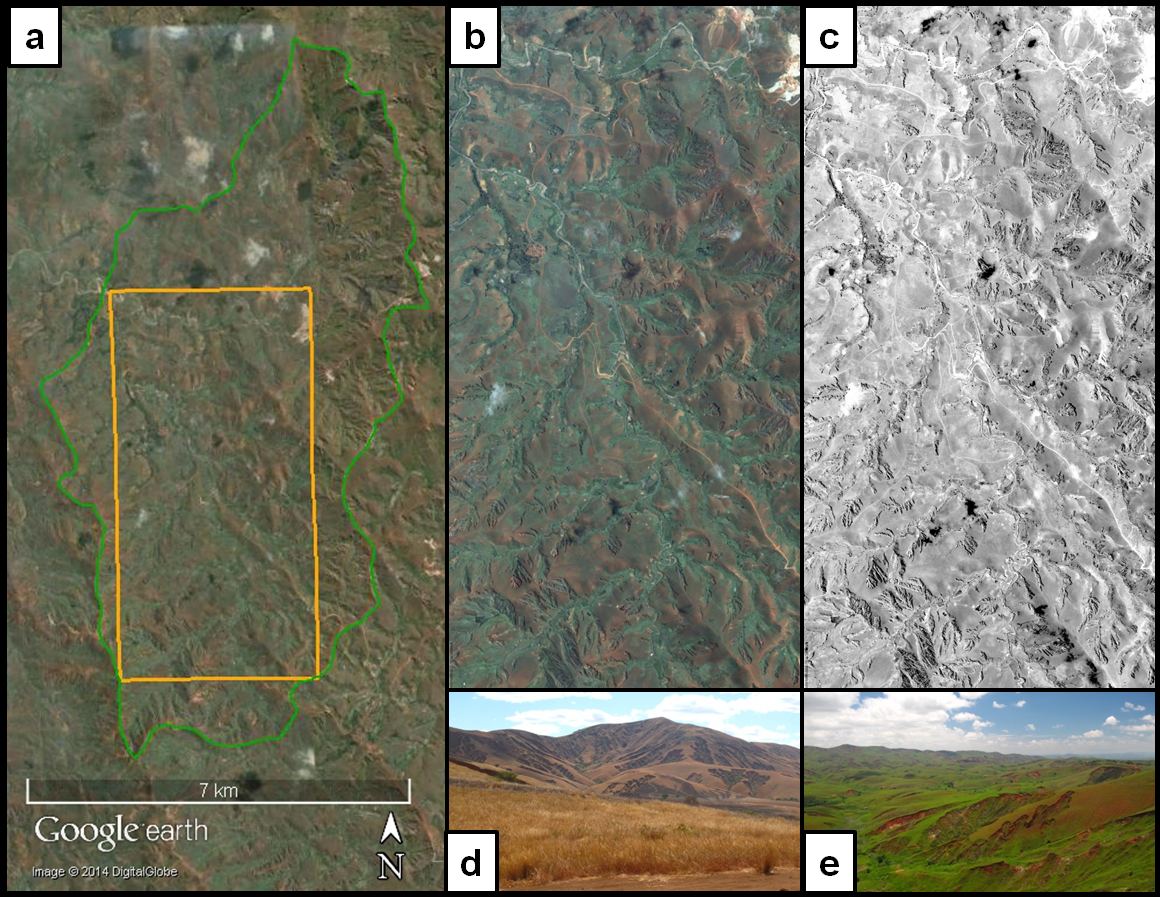

Supplement: Supplementary file 12 — Supplementary material 12 (TIFF 3053 kb) [file 10021_2014_9772_MOESM12_ESM.tiff]
